# Rapid identification of pediatric brain tumors with differential mobility spectrometry

**DOI:** 10.3389/fonc.2024.1352509

**Published:** 2024-04-30

**Authors:** Ilkka Haapala, Anton Rauhameri, Meri Mäkelä, Markus Karjalainen, Anton Kontunen, Markus Mieskolainen, Hannu Haapasalo, Antti Roine, Niku Oksala, Antti Vehkaoja, Joonas Haapasalo, Kristiina Nordfors

**Affiliations:** ^1^ Department of Neurosurgery, Tampere University Hospital, Wellbeing Services County of Pirkanmaa, Tampere, Finland; ^2^ Faculty of Medicine and Health Technology, Tampere University, Tampere, Finland; ^3^ TAYS Cancer Center, Tampere University Hospital, Wellbeing Services County of Pirkanmaa, Tampere, Finland; ^4^ Olfactomics Ltd., Tampere, Finland; ^5^ Fimlab Laboratories Ltd., Tampere, Finland; ^6^ Department of Pediatrics, Tampere University Hospital, Wellbeing Services County of Pirkanmaa, Tampere, Finland

**Keywords:** differential mobility spectrometry, neuro-oncology, pediatric neuro-oncology, pediatric brain tumor, rapid diagnostics

## Abstract

**Introduction:**

Brain tumors are a major source of disease burden in pediatric population, with the most common tumor types being pilocytic astrocytoma, ependymoma and medulloblastoma. In every tumor entity, surgery is the cornerstone of treatment, but the importance of gross-total resection and the corresponding patient prognosis is highly variant. However, real-time identification of pediatric CNS malignancies based on the histology of the frozen sections alone is especially troublesome. We propose a novel method based on differential mobility spectrometry (DMS) analysis for rapid identification of pediatric brain tumors.

**Methods:**

We prospectively obtained tumor samples from 15 pediatric patients (5 pilocytic astrocytomas, 5 ependymomas and 5 medulloblastomas). The samples were cut into 36 smaller specimens that were analyzed with the DMS.

**Results:**

With linear discriminant analysis algorithm, a classification accuracy (CA) of 70% was reached. Additionally, a 75% CA was achieved in a pooled analysis of medulloblastoma vs. gliomas.

**Discussion:**

Our results show that the DMS is able to differentiate most common pediatric brain tumor samples, thus making it a promising additional instrument for real-time brain tumor diagnostics.

## 1 Introduction

Pediatric brain tumors are the most common solid malignancy of childhood and the most common cause of cancer-related death in children (1). In pediatric population, gliomas are the most frequent brain tumors with WHO grade (gr.) 1 pilocytic astrocytomas (PA) and WHO gr. 2-3 ependymomas being the most common histology. Other notable tumor subgroups include embryonal tumors with medulloblastoma being the most frequent one. Another notable malignant tumor subgroup is diffuse midline glioma, which histologically may appear as low-grade, but molecular testing reveals the characteristic H3K27 mutation in the histone 3 protein as a sign for higher malignancy (2). The complete list of all tumor entities is numerous and the accurate division of them varies significantly across age groups (3).

Generally, the cornerstone of treatment for pediatric brain tumors includes surgical resection if possible and chemotherapy with or without radiation as appropriate (1). The role of surgery is highlighted in circumscribed tumors, such as PAs and ependymomas, where the extent of surgical resection is a critical determinant of the outcome. In PAs, a gross-total surgical resection alone with a sparse follow-up imaging is considered a sufficient treatment with an excellent prognosis (4). For ependymomas, a gross-total resection is associated with the lowest rates of mortality, the best overall survival, and the longest progression-free survival regardless of tumor location (5).

Pathological grade does not have much prognostic relevance for ependymomas in general and genetic subgrouping is better correlated with survival (6). With DNA methylation analysis, posterior fossa ependymomas can be divided into subgroups A (PFA) and B (PFB) with group A occurring chiefly in early childhood and being very aggressive (7). Supratentorial ependymomas are genetically different disease with more than 70% of them carrying a fusion in the RELA -gene (8).

Medulloblastoma is the most common malignant brain tumor in the pediatric population. Genetically, medulloblastomas are divided into four subgroups of different levels of aggressiveness and variant patient prognosis: wingless and INT-1 (WNT)-activated, sonic hedgehog (SHH)-activated, group 3 and group 4 (9). The last two groups are often combined as non-WNT/non-SHH medulloblastomas due to genetic overlapping and similar clinical behavior. For medulloblastomas, the benefit of gross-total resection is less clear than for pilocytic astrocytomas or ependymomas. According to the most recent study on the issue, a nearly-total resection (residual tumor less than 1,5 cubic centimeters) of the tumor leads to the same prognosis as gross-total resection regardless of the medulloblastoma subgroup (10).

Currently, frozen section analysis is the gold standard for intraoperative tissue identification. However, identifying a pediatric CNS tumor is considered to be especially troublesome. The pathologists are generally advised to offer unspecific diagnostic categories instead of accurate subtypes and to defer grading (11). In some cases, as in medulloblastomas or posterior fossa ependymomas, subtype identification using nothing but the histological appearance of frozen section is considered impossible. This directly calls for new solutions.

Differential mobility spectrometry (DMS) is a modality that characterizes substances by the mobility differences of ionized particles in high-frequency electrical fields, which results in a substance-specific dispersion spectrum, or “smell fingerprint” (12). The function of the DMS is somewhat analogous with mass spectrometry. However, the DMS is a much smaller and cheaper device that is easier to operate and maintain and has a very low detection limit and the capability for analysis in almost real-time. Also, the analysis can be made of very small tissue samples with only 1 mm^3^ required volume minimum to produce adequate DMS signal (13, 14).

We have previously shown that the DMS is able to identify different brain tumors and injured brain tissue ex vivo and classify glioma samples based on their IDH -mutation status (13, 15). In this study we investigated its ability to perform rapid and preparation-free identification of different pediatric brain tumors: pilocytic astrocytomas, ependymomas and medulloblastomas.

## 2 Materials and methods

We prospectively obtained samples from 15 pediatric brain tumor patients that were operated in Tampere University Hospital, Finland, between the years 2013-2022. 13 tumors were previously untreated (primary) and 2 were recurrent. The tumors included 5 ependymomas, 5 pilocytic astrocytomas and 5 medulloblastomas. In addition to histology and immunostaining, the utilized diagnostic tests included fluorescence *in situ* hybridization (FISH), next generation sequencing (NGS) and DNA methylation analysis. Out of the five medulloblastomas, two were SHH -activated, TP53 wild type, one was SHH -activated, TP53 undefined and the remaining two were classified as non-SHH/non-WNT medulloblastomas genetically. Both infratentorial ependymomas were genetically PFAs (posterior fossa group A). One supratentorial ependymoma had a RELA-fusion. However, in the two other supratentorial ependymomas, there was a substantial incongruity between the histology and the genetic profile with one being classified as CNS neuroblastoma and the other as PA by genetics. All cases were carefully reviewed in tumor board with experienced neuropathologists and in both cases of incongruency between histology and genetic results the decision was made to adhere to histology. In the PA group, only one infratentorial and one supratentorial tumor underwent genetic testing and both were well-matched to their histology and location. Patient characteristics and the utilized diagnostic methods are presented in **Table 1**. A detailed description of the diagnostic tests and their results tumor wise is presented in a **Supplementary Table**.

**Table 1:** Patient characteristics.

	Medulloblastoma	Ependymoma	PA
**Age (mean ± S.D., years)**	7,1 ± 5,6	4,6 ± 3,0	7,7 ± 6,9
**Females**	2	2	2
**Males**	3	3	3
Location			
Infratentorial	5	2	4
Supratentorial	–	3	1
**Primary**	4	5	4
**Recurrent**	1	–	1
Genetics			
Infratentorial	2 SHH (TP53 wild type), 1 SHH (TP53 undefined), 2 non-SHH/non-WNT	2 PFA	2 PA-INF, 2 untested
Supratentorial	–	1 ST-RELA, 2 misidentified	1 PA-CORT
**Methods for genetic/epigenetic testing**	DNA methylation analysis, FISH, NGS	DNA methylation analysis	DNA methylation analysis

PA, pilocytic astrocytoma; SHH, sonic hedgehog; WNT, wingless and INT; PFA, posterior fossa group A; ST-RELA, supratentorial with RELA-fusion; PA-INF, infratentorial pilocytic astrocytoma; PA-CORT, cortical pilocytic astrocytoma; FISH, fluorescence in situ hybridization; NGS, next generation sequencing.

**Figure 1:**
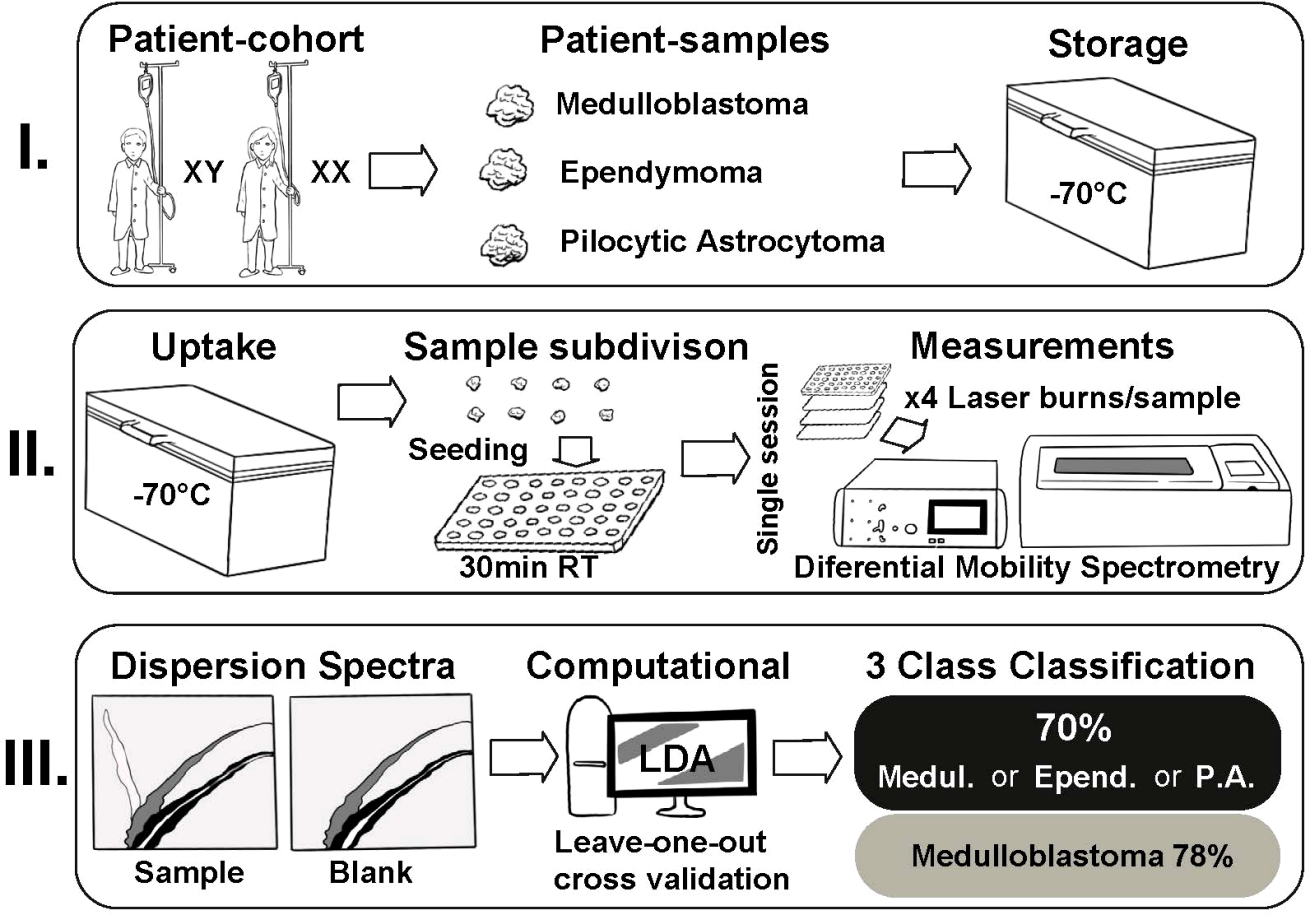
The experiment workflow.

All the samples were stored in a freezer at -70°C. The samples were meticulously cut into 36 smaller specimens of roughly equal sizes. Due to the different sizes of the original samples, the final set of specimens included 17 medulloblastomas, 13 ependymomas and 6 pilocytic astrocytomas. Blood and visual necrosis due to surgical electrocoagulation were removed from samples prior to the analysis. 36 samples were randomly placed in four plastic well plates with each well containing 0.18 mL of agar in the bottom. The samples were seated 30 minutes prior to the start of the measurement for thawing. The well plates were photographed after seating. Each sample was incised with a custom-built, computer-controlled, 40 W, 10.6 μm CO_2_ laser evaporator ATLAS (Olfactomics Ltd., Finland) four times in a quadratic manner, with 1 mm gaps between the incisions. The duration of the measurements (from laser burn to achieving DMS signal) was 12-13 seconds with 60 seconds waiting time between subsequent measurements. The total number of incisions was 144. Before and after the incisions, reference measurements were taken of empty wells for system calibration and quality control purposes. After the measurements, another photograph was taken of the well plate to control the accuracy and quality of the laser marks in the samples. All four well plates were analyzed successively in a single session. A schematic illustration of the experiment workflow is presented in **Figure 1** and detailed in **Supplementary 1**.

**Figure 2:**
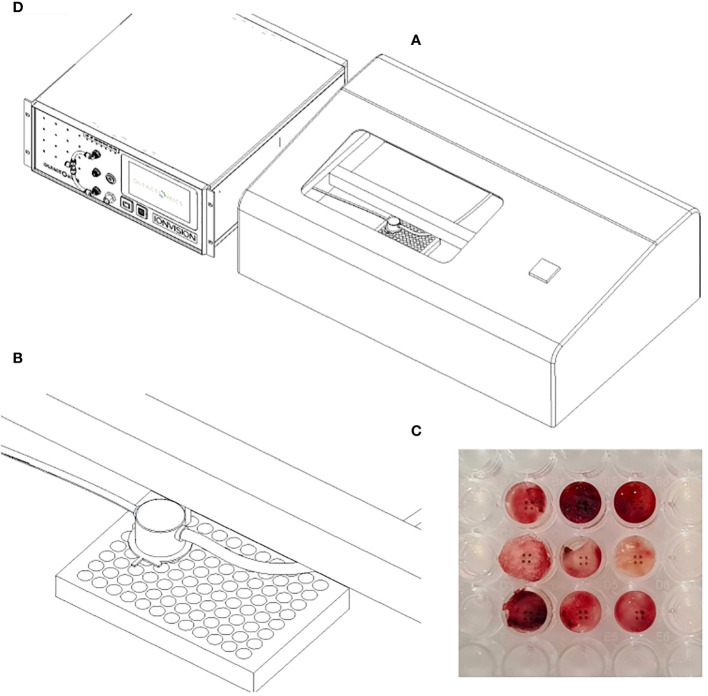
The setup for DMS analysis: **(A)** Laser sampling unit (ATLAS). A custom-built metallic box in which the computer-controlled laser nozzle for electrocoagulation and a suction tube for evacuating the resulting smoke moves above the well plate **(B)** Enlargement of the laser nozzle and suction tube inside the sampling unit **(C)** Signs of laser coagulation in tumor specimens after sampling **(D)** DMS analyzer (IonVision) where the smoke from ATLAS is ionized and driven into the sensor.

The resulting smoke was transported to the DMS inlet with purified and humidified pressurized air used as a carrier gas. The DMS used in the study was a commercial IonVision instrument (Olfactomics Ltd., Finland). In DMS, the sample molecules were ionized and transported into a sensor unit which separated the ions with low and high electric fields. In the altering electric fields, the clustering-declustering behavior of the sample ions resulted in a characteristic two-dimensional dispersion spectrum (“smell fingerprint”) that was used for identification and classification purposes. An illustration of ATLAS and IonVision is presented in **Figure 2**.

Since the visual differences in the dispersion spectra were subtle, the classifications were made by using a machine learning algorithm. Several different algorithms were tested, and the best performing algorithm was found to be linear discriminant analysis (LDA). The algorithm was first taught about the data features with a training data set, which was a set of know objects. The function of the algorithm was then tested with a test set where the algorithm had to assign the class. Leave-one-group-out cross validation was used to avoid overfitting, which means the algorithm making its classification decisions not based on the actual features of the class, but rather on variance or background noise of the particular training set in use. The study was approved by the ethics review board of Pirkanmaa Hospital District, Finland. The written consent for the study was obtained either from the patient or parent as appropriate.

## 3 Results

**Figure 3:**
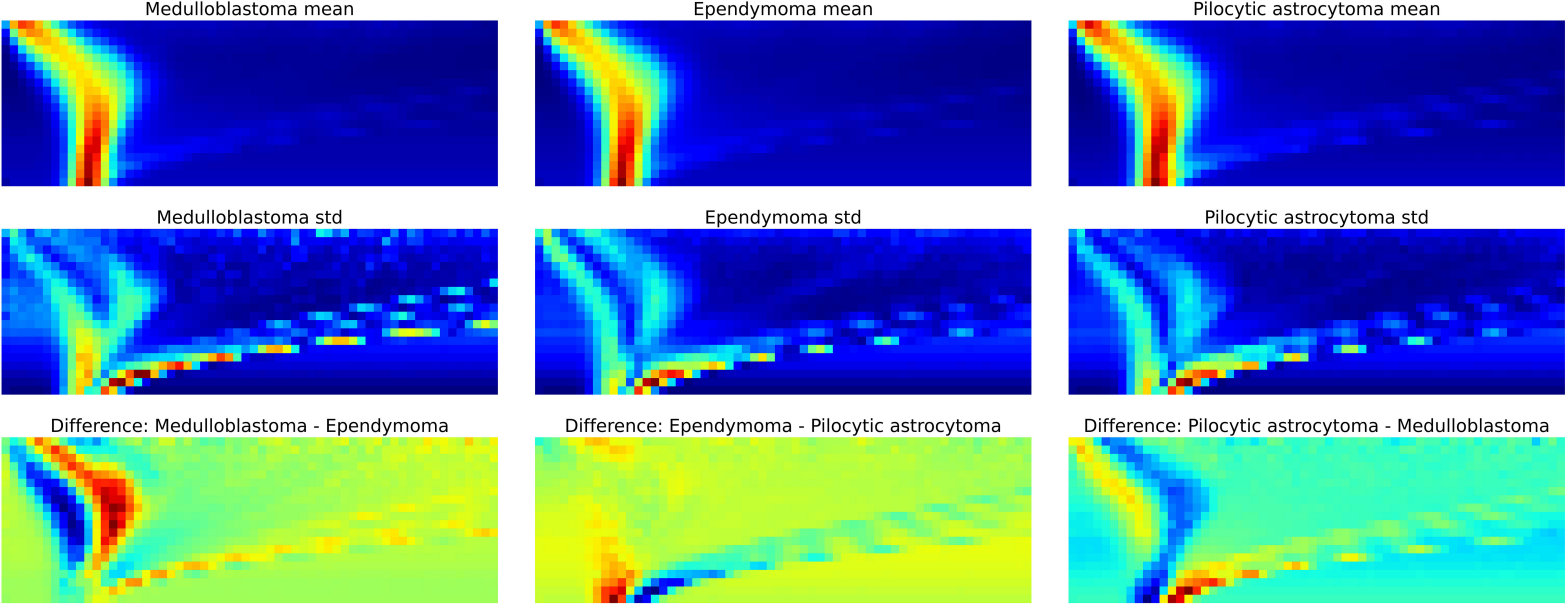
The dispersion spectra. Top row: the average dispersion spectra of each class calculated pixel wise. Middle row: the variation in the dispersion spectra within each class. Bottom row: the differences in the spectra between classes. The red color indicates greater values for the former and blue color for the latter tumor group in the given binary classification (for example in the bottom left comparison: former – medulloblastoma, latter – ependymoma).

With the LDA algorithm and leave-one-group-out cross validation, the overall classification accuracy (CA, percentage of samples correctly classified) for 3-class classification was 70% (**Table 2**). The algorithm was best able to classify medulloblastoma samples with 78% of the specimens correctly classified. Ependymomas and PAs proved to be more difficult to classify as only 58% of ependymomas and 54% of PAs were accurately classified. The mean spectra of the three tumor classes and their differences are shown in **Figure 3**. The spectra are presented as colored heat maps, where every pixel represents a measured detector response with certain values of radio-frequency voltage waveform (Y-axis) and direct current compensation voltage (X-axis). The top row shows the mean spectra of each tumor class, and the middle row presents the standard deviation within each class pixel wise. The bottom row shows the differences between classes with red color indicating greater values for the former and blue color for the latter tumor group in the given binary comparison. In the spectra, ion peaks on the left with low compensation voltage were the most prone to exhibit the differences between the tumor tissues.

**Table 2:** Classification results of the 3-class classification.

	Assigned class				Sens.	Spec.	CA
**True class**	**Medulloblastoma**	**78%**	21%	8%	78%	81%	**70%**
	**Ependymoma**	17%	**58%**	38%	69%	70%	
	**PA**	5%	21%	**54%**	30%	95%	
		**Medulloblastoma**	**Ependymoma**	**PA**			

sens., sensitivity; spec., specificity; CA, classification accuracy.

Values in bold indicate the percentages of the measurements of each tumor class assigned correctly and the general classification accuracy.

**Figure 4:**
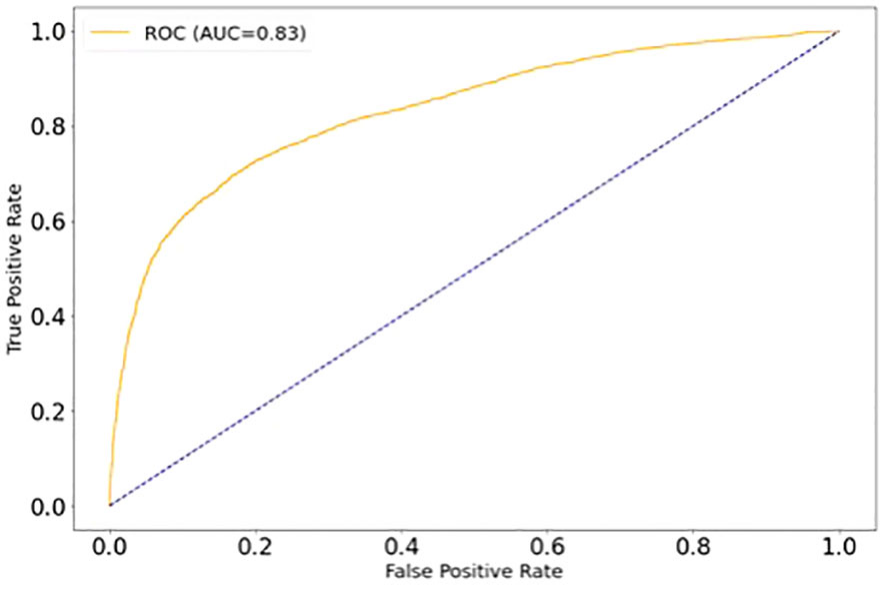
ROC -curve of the binary classification (medulloblastoma vs. gliomas).

Additionally, we pooled the gliomas (pilocytic astrocytomas and ependymomas) together to make a binary classification with more material to adequately train the algorithm and to achieve better-balanced classes. With the LDA algorithm and leave-one-group-out cross validation, the overall CA for the classification was 75%, with 73% of medulloblastoma samples and 77% of glioma samples correctly classified (**Table 3**). ROC -curve of the binary classification is presented in **Figure 4** with area under curve (AUC)=0.83. The classification results of individual samples are presented in **Supplementary 2**. A thorough presentation of the data analysis and the results is provided in **Supplementary 3**.

**Table 3:** Classification results of the binary classification.

	Assigned class			Sens.	Spec.	CA
**True class**	**Medulloblastoma**	**73%**	23%	73%	77%	**75%**
	**Gliomas**	27%	**77%**			
		**Medulloblastoma**	**Gliomas**			

sens., sensitivity; spec., specificity; CA, classification accuracy.

Values in bold indicate the percentages of the measurements of each tumor class assigned correctly and the general classification accuracy.

## 4 Discussion

Our results show that the DMS is able to identify three of the most common pediatric brain tumors (pilocytic astrocytomas, ependymomas and medulloblastomas) with good accuracy. Also, gliomas as a whole can be discriminated from medulloblastomas. The importance of gross-total resection differs in gliomas compared with medulloblastomas. Therefore, the accurate and real-time intraoperative categorization of the tumors would help the surgeon to tailor the resection strategy. The experiment setup is compact and can be easily fitted into standard neurosurgical operating theater for intraoperative use without further modification.

Generally, the advantages of the DMS compared with other emerging tissue identification solutions (mass spectrometry, Raman spectroscopy, scanning electron microscopy etc.) include small size, an affordable price, relatively low maintenance and ease of operation (13, 14).

We believe that decisive factors in the differentiation of tumor entities are the differences in the metabolic and proteomic profiles of gliomas and embryonal tumors. Since the DMS analysis is based on pattern recognition of smell fingerprints instead of explicit information of the substance molecules, the exact differentiating factor cannot be reliably recognized.

In medulloblastomas and ependymomas, several clinically relevant subtypes have been identified in molecular characterization that considerably affect the patient’s prognosis. Subgroup identification was a matter of great interest but unfortunately, we didn’t have enough sample material to train the classifier for reliable subtype identification.

Several issues related to the experiment setup can explain the shortcomings in the obtained classification accuracy. In medulloblastomas and ependymomas, the metabolic profiles of the tumor cells vary between subtypes, thus introducing heterogeneity to the classes. However, these assumed differences of the DMS spectra of tumor subtypes cannot be directly shown in our material due to inadequate number of samples. Albeit the number of the original tumor samples was even, medulloblastoma samples were bigger and provided more smaller specimens, so eventually the material became skewed. This disturbed the classification by making the classifier to favor the bigger class, and the resulting bias could only partially be controlled with computational methods. In binary classification, the classes were better balanced that could also partially explain the better classification accuracy.

Additionally, even as we collected the samples over a 10-year period of time, the overall number of samples was not sufficient to optimally train the algorithm even for 3-class-classification due to the rarity of the tumor entities. Algorithm training means that the classifier is first taught about the data features with training data set and the function of the algorithm is then tested with a test set, in which the algorithm has to assign the class (16). A small training data set increases the risk of overfitting, which means that the algorithm makes its classification decisions not based on the actual features of the class, but rather on variance or background noise of the particular training set in use. To test the reliability of the data, we performed a leave-one-group-out cross validation. In that method, the data set is first divided into as many subsets as it can be. Then the algorithm is tested by using every single item as test set, while every other sample act as training set (17).

Moreover, even with our best preparation of the samples, the individual sizes of the samples were somewhat variant. This inevitably affected the DMS signal strength and despite data normalization, has additionally confounded the classifier. On the other hand, keeping the sample preparation at the absolute minimum is essential for the fluency of the workflow of the intended intraoperative use. Also, years of freezing may have blurred some of the DMS-detectable elements due to sample denaturation.

Most of the challenges of this study could be overcome by simply increasing the sample size. It would allow a more reliable training of the classifier and provide access to explore subtype-level identification of the tumors. This will require collaboration to achieve sufficient amounts of sample material of these relatively rare tumor entities. Also, the acquisition of the samples should be done in a prospective manner for up-to-date genetic information.

## 5 Conclusions

Our results show that the DMS is able to identify three of the most common pediatric brain tumors with good accuracy. Potentially, the DMS could become an additional tool for intraoperative pediatric brain tumor diagnostics and tailoring the surgical resection strategy. Larger cohorts are needed to explore the identification capability of the DMS in tumor subtypes and for building more robust and high-performing classifiers.

## Data availability statement

The original contributions presented in the study are included in the article/**Supplementary Material**. Further inquiries can be directed to the corresponding author.

## Ethics statement

The studies involving humans were approved by The Ethics Review Board of Pirkanmaa Wellbeing District, Finland. The studies were conducted in accordance with the local legislation and institutional requirements. The human samples used in this study were acquired from primarily isolated as part of your previous study for which ethical approval was obtained. Written informed consent for participation was not required from the participants or the participants’ legal guardians/next of kin in accordance with the national legislation and institutional requirements.

## Author contributions

IH: Writing – review & editing, Writing – original draft, Methodology, Investigation, Conceptualization. ARa: Writing – review & editing, Validation, Software, Formal analysis, Data curation. MMä: Writing – review & editing, Validation, Software, Investigation, Formal analysis, Data curation. MK: Writing – review & editing, Visualization, Supervision, Software, Investigation, Data curation. AK: Writing – review & editing, Supervision, Software, Methodology, Investigation. MMi: Writing – review & editing, Investigation. HH: Writing – review & editing, Supervision, Resources, Project administration, Methodology, Investigation, Conceptualization. ARo: Writing – review & editing, Supervision, Resources, Project administration, Methodology, Investigation, Conceptualization. NO: Writing – review & editing, Supervision, Resources, Project administration, Conceptualization. AV: Writing – review & editing, Supervision, Project administration, Formal analysis, Data curation, Conceptualization. JH: Writing – review & editing, Validation, Supervision, Resources, Project administration, Methodology, Funding acquisition, Conceptualization. KN: Writing – review & editing, Supervision, Resources, Project administration, Methodology, Funding acquisition, Conceptualization.
