# Supplementary material for: Rapid identification of pediatric brain tumors with differential mobility spectrometry
Source: Front Oncol. 2024 Apr 30;14:1352509. doi: 10.3389/fonc.2024.1352509 (PMC11091372; doi:10.3389/fonc.2024.1352509)
Supplement: Supplementary file 1 [file DataSheet_1.pdf]

July 11, 2022

Measurements with paediatric brain cancers

SUMMARY

This document outlines the schedule and protocol for the measurements of paediatric brain cancers on July 12th, 2022.

1 MEASUREMENT SYSTEM: PREPARATION AND PARAMETERS

Due to contamination issues, the measurements had to be postponed from 6.7. to 12.7.

In 11.7., the reference spectrum was adequately clean, and the following parameters were set for the measurement with the ATLAS system:

Ionvision settings:
- Project: Laser
- Parameter preset: Laser 0.75 sample (or Laser)*

Laser settings:
- Number of X points: 3
- Number of Y points: 3
- Sub X points: 2
- Sub Y points: 2
- Laser pulse length: 2 ms
- Pulse repetition time: 48 ms
- Number of pulses: 250
- Waiting time before measurement: 1 s
- Waiting time after measurement: 60 s*

*The parameter preset can be changed after the measurement of well plate 1, if needed.

The system and parameters are also depicted in Figure 1.

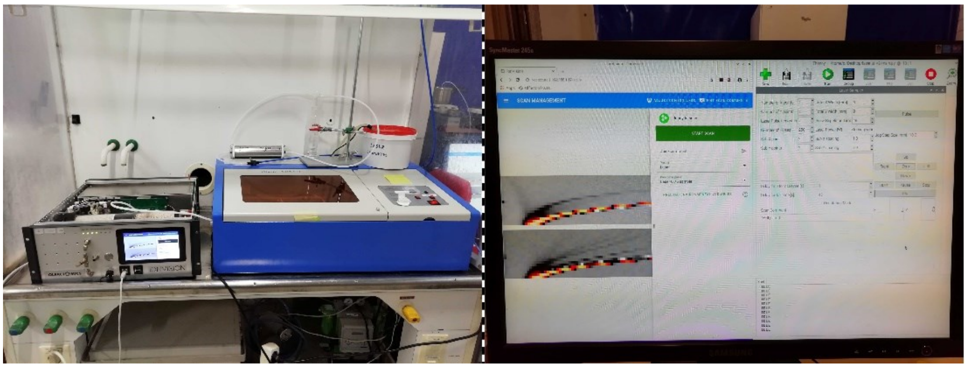

Figure 1. System and parameters

2 SCHEDULE

All samples will be measured during 12.7. The preliminary schedule is presented below:

Time	Task
6.00	System ON to ensure that any contamination that has potentially diffused within the system during the night will not impede the measurements
7.30	Gathering of the samples from TAYS pathology and delivery to Hervanta
8.30	Preparation of the first well plate
9.00-10.00	Measurement of the first sample and the first well plate (10 samples in total, 40 spectra)
10.30	Preparation of the second well plate
11.00-12.00	Measurement of the second well plate (9 samples in total 36 spectra)
12.30	Preparation of the third well plate
13.00-14.00	Measurement of the third well plate (9 samples in total 36 spectra)
14.30	Preparation of the fourth well plate
15.00-16.00	Measurement of the third well plate (8 samples in total 32 spectra)
16.30	System OFF, end of measurement day

3 PROTOCOL

The measurement day will consist of preparation and measurement of four well plates. The well plates have a small amount of agar in some of the wells to elevate the bottom of the wells. The total number of cancer samples is 36. All of these samples will be measured 4 times, totaling 144 measurements. The samples will be measured in 3X3 matrices. Small deviations will be made in plates 1 and 4. The protocol is as follows:

1. The first well plate
    a. **Place the samples on the well plate 30 minutes before the start of the measurement series to ensure that the samples are thawed**
    b. There will be 10 samples out of which the first will function as a test sample to ensure that the parameters are feasible. The default parameter preset will be "Laser 0.75 sample" but can be switched to "Laser" if the signals are too low.
    c. The placement of the samples is depicted in Figure 2.

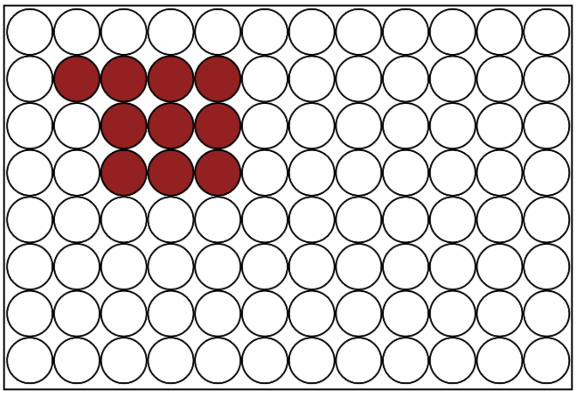

Figure 2. Well plate 1

    d. After placing the samples in the well plate, take a photograph
    e. Calibrate the position of the nozzle
    f. **Before starting, take a reference measurement manually. The comment can be "Reference measurement before Well plate 1"**
    g. The measurement of the well plate will start. The comment in the laser UI will be "Well plate 1"
    h. **After the measurement is over, take a reference measurement manually. The comment can be "Reference measurement after Well plate 1"**
    i. Photograph the well plate

2. The second well plate
    a. **Place the samples on the well plate 30 minutes before the start of the measurement series to ensure that the samples are thawed**
    b. There will be 9 samples. Use the same parameters as with Well plate 1.
    c. The placement of the samples is depicted in Figure 3.

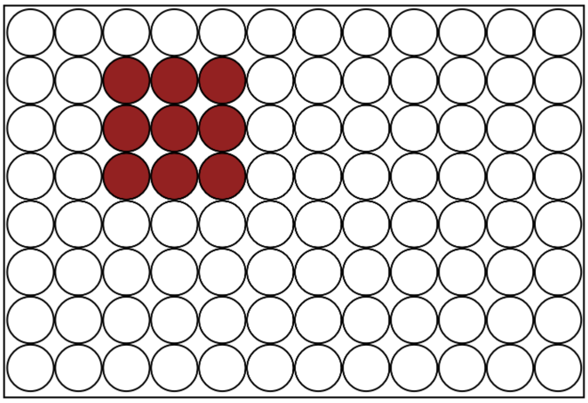

Figure 3. Well plate 2

    d. After placing the samples in the well plate, take a photograph
    e. Calibrate the position of the nozzle
    f. **Before starting, take a reference measurement manually. The comment can be "Reference measurement before Well plate 2"**
    g. The measurement of the well plate will start. The comment in the laser UI will be "Well plate 2"
    h. **After the measurement is over, take a reference measurement manually. The comment can be "Reference measurement after Well plate 2"**
    i. Photograph the well plate
3. The third well plate
    a. **Place the samples on the well plate 30 minutes before the start of the measurement series to ensure that the samples are thawed**
    b. There will be 9 samples. Use the same parameters as with Well plate 1.
    c. The placement of the samples is depicted in Figure 4.

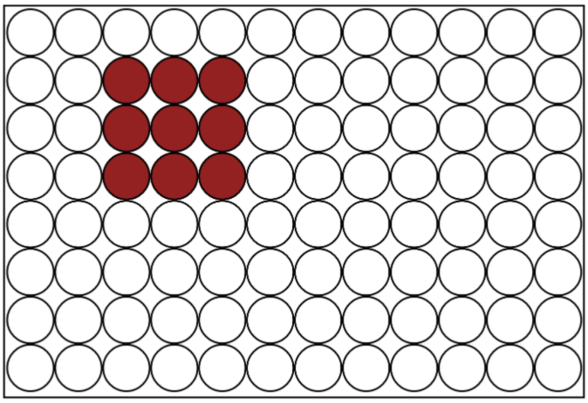

Figure 4. Well plate 3

    d. After placing the samples in the well plate, take a photograph
    e. Calibrate the position of the nozzle
    f. **Before starting, take a reference measurement manually. The comment can be "Reference measurement before Well plate 3"**
    g. The measurement of the well plate will start. The comment in the laser UI will be "Well plate 3"
    h. **After the measurement is over, take a reference measurement manually. The comment can be "Reference measurement after Well plate 3"**
    i. Photograph the well plate

4. The fourth well plate
    a. **Place the samples on the well plate 30 minutes before the start of the measurement series to ensure that the samples are thawed**
    b. There will be 8 samples. The final well in the 3X3 matrix can be filled with water. Use the same parameters as with Well plate 1.
    c. The placement of the samples is depicted in Figure 5.

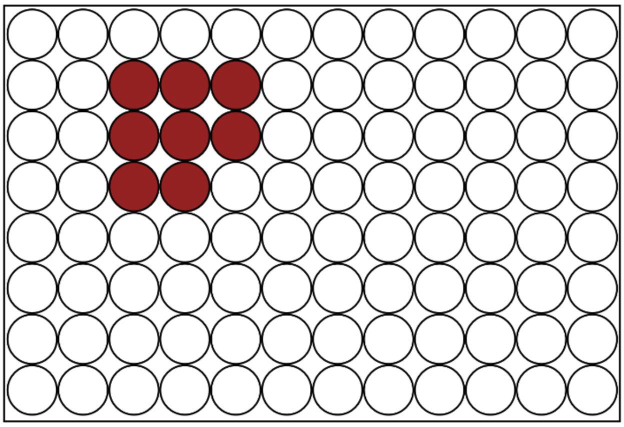

Figure 5. Well plate 4

    d. After placing the samples in the well plate, take a photograph
    e. Calibrate the position of the nozzle
    f. **Before starting, take a reference measurement manually. The comment can be "Reference measurement before Well plate 4"**
    g. The measurement of the well plate will start. The comment in the laser UI will be "Well plate 4"
    h. **After the measurement is over, take a reference measurement manually. The comment can be "Reference measurement after Well plate 4"**
    i. Photograph the well plate
