# Supplementary material for: Rapid identification of pediatric brain tumors with differential mobility spectrometry
Source: Front Oncol. 2024 Apr 30;14:1352509. doi: 10.3389/fonc.2024.1352509 (PMC11091372; doi:10.3389/fonc.2024.1352509)
Supplement: Supplementary file 2 [file DataSheet_2.pdf]

Supplementary information

January 24, 2023

1 1. Introduction

This document provides supplementary information for the article by Haapala et. al. on utilized data processing approaches for Differential Mobility Spectrometry dispersion plots.

2 Load libraries needed for analysis and classification, defining global parameters

[27]:
```
import numpy as np
import pandas as pd
import matplotlib.pyplot as plt
import matplotlib.image
from scipy.signal import detrend
import os
import json
import pickle as pkl
import matplotlib.gridspec as gridspec
import statsmodels.api as sm
from scipy.stats import linregress
gticks_fontsize = 15
gtitles_fontsize = 25
gaxislabels_fontsize=20
gfigsize = (15,5)
%matplotlib inline
```

3 Analysis of environmental factors

In this section analysis of the environmental factors is provided. The environmental factors, such as temperature and humidity, could possibly affect the measurements bringing bias into the data. For example, it is known that the response of DMS is inversely proportional to the moisture level [1].

[32]:
```
plt.figure(figsize=(20,10))
implot = np.flipud(data.iloc[0]['intensity_top'].reshape(20,60))
implot = np.apply_along_axis(lambda row: (row - np.mean(row))/np.std(row), 1,␣
 ↪implot)
plt.imshow(implot)
```

```
[32]: <matplotlib.image.AxesImage at 0x7fed7d6bda60>
```

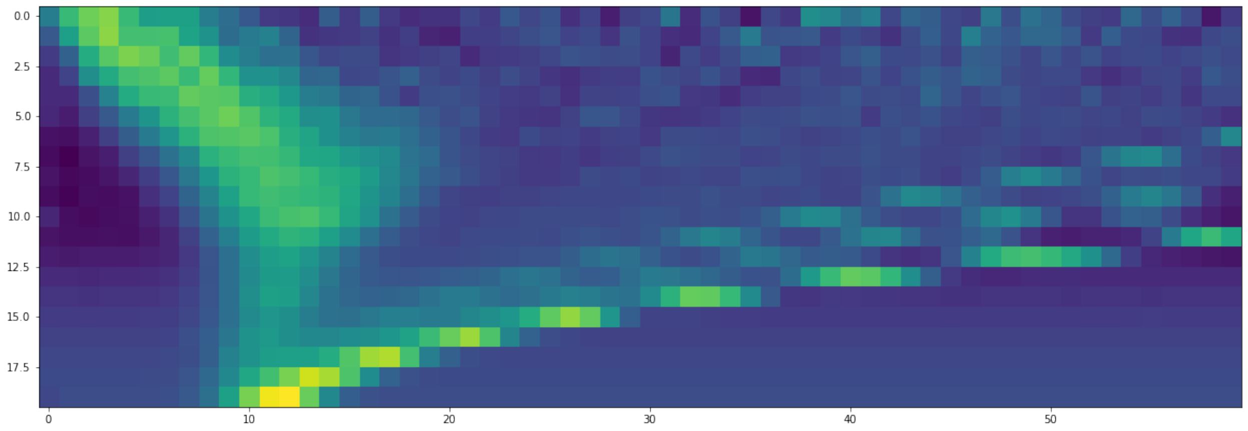

Figure 1: Example dispersion plot of Medulloblastoma

3.1 Temperature

```
[33]: plt.figure(figsize=(15,5))
plt.plot(data['sample_temperature'].to_list())
_ = plt.axvline(39, color='red')
_ = plt.axvline(39+36, color='red')
_ = plt.axvline(39+36*2, color='red')
_ = plt.xticks(fontsize=gticks_fontsize)
_ = plt.yticks(fontsize=gticks_fontsize)
plt.xlabel("Samples", fontsize=gaxislabels_fontsize)
plt.ylabel("Tempereature [$^\circ$C]", fontsize=gaxislabels_fontsize)
plt.xlim([-1, 139])
for i, j in enumerate([10, 50, 85, 120]):
    _ = plt.text(j,26.3,f'well plate {i+1}', fontsize=15)
tempX = np.arange(len(data))
res = linregress(tempX, data['sample_temperature'])
print(f"Fitting linear model to the temperature. Intercept: {res.intercept:.2f}, ␣
↪slope: {res.slope:.3f}")
```

```
Fitting linear model to the temperature. Intercept: 25.94, slope: 0.010
```

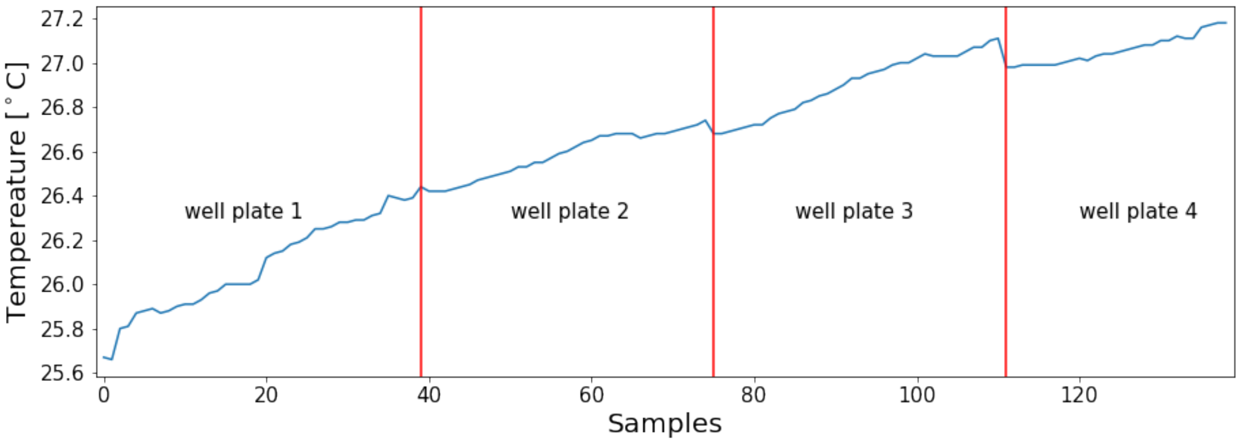

Figure 2: Temperature change during the measurements

For the analyzed dispersion plot the temperature plot (Figure 2) demonstrates a clear upward trend. During the measurements the temperature increased from 26.6 $°C$ to 27.2 $°C$. The fitted linear model suggests that the temperature changed on average 0.01 degree/sample.

The red vertical lines on the plot above divide data by well plates. Figure 3 shows an example of the well plates used in the study.

The samples were placed into such well plates and exposed to an evaporation system. There were 4 well plates during measurements.

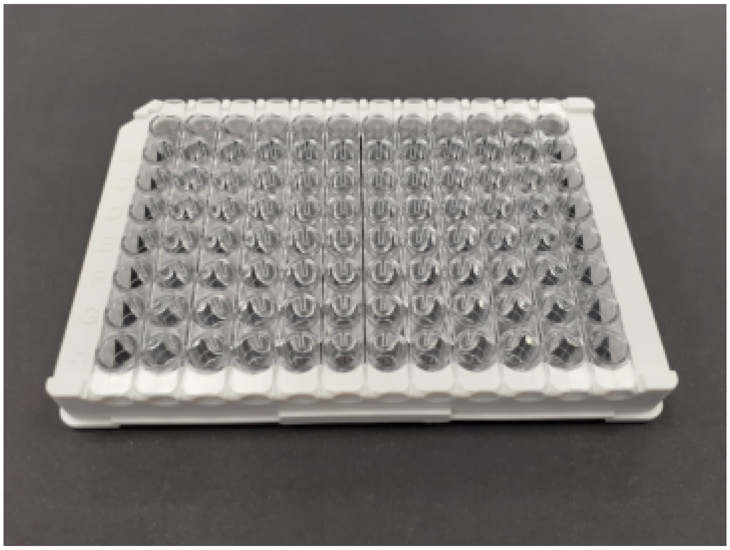

Figure 3: Example well plate used in laboratories

Let us see if there is a general trend in the pixels of the dispersion plots. The trend analysis is aimed at revealing if there is accumulation of VOCs in the system or bias in the measurements caused by environmental factors. We stack all the dispersion plots as in Figure 4 (sorted by time) and fit linear models to each set of pixels (combination of compensation voltage and separation voltage).

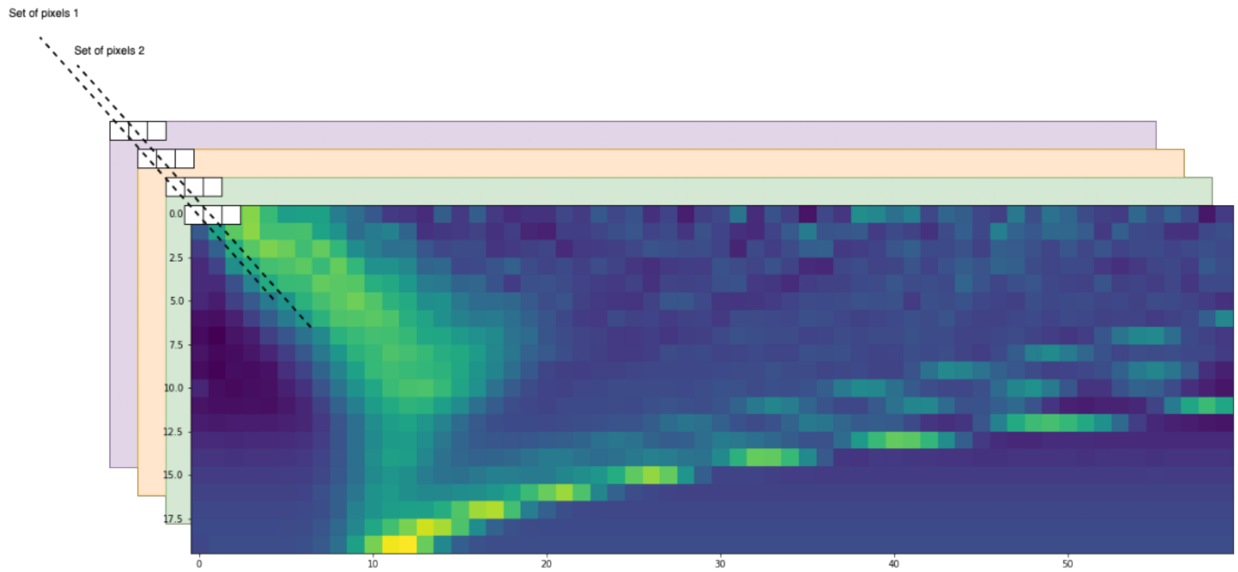

Figure 4: Visualization of the stacked dispersion plots.

[9]:
```
tempX = np.array(data['intensity_top'].to_list()) # extract all "intensity_top"␣
↪vectors from the dataframe
dpX = np.zeros((len(data), 20, 60)) # prepare an empty matrix for stacking the␣
↪dispersion plots
for i in range(len(data)): # loop over each "intensity_top"
    sample = np.flipud(np.array(tempX[i,:]).reshape(20, 60)) # extract and␣
↪reshape "intensity_top" vector
    dpX[i,:,:] = sample # put into the dpX matrix

slopes = [] # list for collecting slopes for further analysis
for i in range(dpX.shape[1]): # loop over compensation voltages (x-axis on a␣
↪dispersion plot)
    for j in range(dpX.shape[2]): # loop over separation voltages (y-axis on a␣
↪dispersion plot)
        py = dpX[:,i,j] # select pixel set (as on image above)
        px = np.arange(len(data)) # prepare x-values (1,2,3...) for fitting␣
↪linear model
        res = linregress(px, py) # perform linear regression
        slopes.append(res.slope) # extract slope
print(f"Median slope: {np.median(slopes):.3f}, std: {np.std(slopes):.3f}")
_ = plt.hist(slopes)
_ = plt.title('Distribution of trends in the data')
```

```
Median slope: 0.000, std: 0.070
```

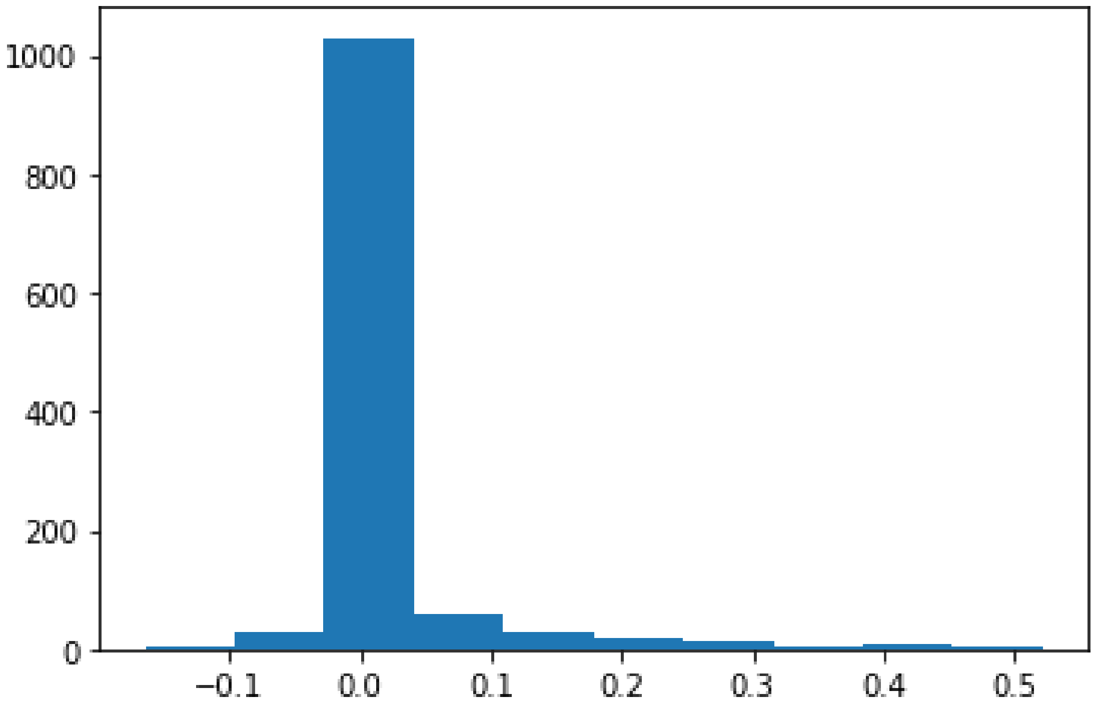

Figure 5: Distribution of trends in the data

The histogram in Figure 5 shows distribution of trends over the whole data set (sorted by time and stacked). As explained above, a linear model is fitted to a pixel set and the trend is extracted. Since each dispersion plot contains 1200 pixels, the bar plot contains 1200 values of trends. It can be seen from the plot that out of 1200 slopes 1029 slopes fall in range of -0.027 and 0.041. The remaining 171 slopes fall in range [-0.164, -0.027] and [0.041, 0.52].

Figure 6 shows distribution of the trends on the dispersion plot. The red area contains slopes that are in the range (0.3, 0.52]. The yellow area contains slopes that are in (0.1, 0.3]. The green area contains slopes that are less or equal to 0.1. The upper part and the part on the right of the yellow area on the dispersion plot is usually the area where the separations are observed. The image shows that the trends in this area are close to zero. The trends in the red and yellow areas are negligible because range of values in the dispersion plots is commonly, but not limited to, ±300 pA, meaning that slopes of up to 0.52 are no reason for concern.

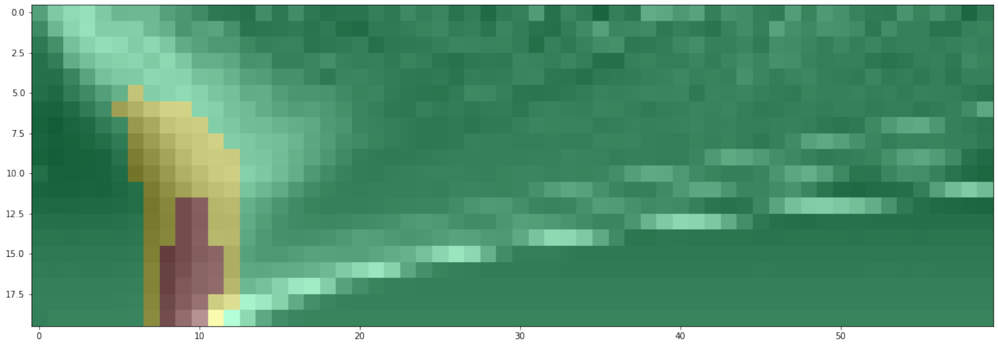

Figure 6: Distribution of slopes on the dispersion plot

The analysis shows that on average, intensities do not demonstrate tendency to grow with the temperature.

4 Humidity change sorted by time

[10]:

```
plt.figure(figsize=gfigsize)
plt.plot(data['sample_humidity'].to_list())
_ = plt.title("Humidity change during measurement.", fontsize=gtitles_fontsize)
_ = plt.axvline(39, color='red')
_ = plt.axvline(39+36, color='red')
_ = plt.axvline(39+36*2, color='red')
_ = plt.xticks(fontsize=gticks_fontsize)
_ = plt.yticks(fontsize=gticks_fontsize)
_ = plt.xlabel("Samples", fontsize=gaxislabels_fontsize)
_ = plt.ylabel("Relative humidity [%]", fontsize=gaxislabels_fontsize)
print('Maximum humidity value: ', data['sample_humidity'].max())
print('Minimum humidity value: ', data['sample_humidity'].min())
```

```
Maximum humidity value:  15.33
Minimum humidity value:  13.77
```

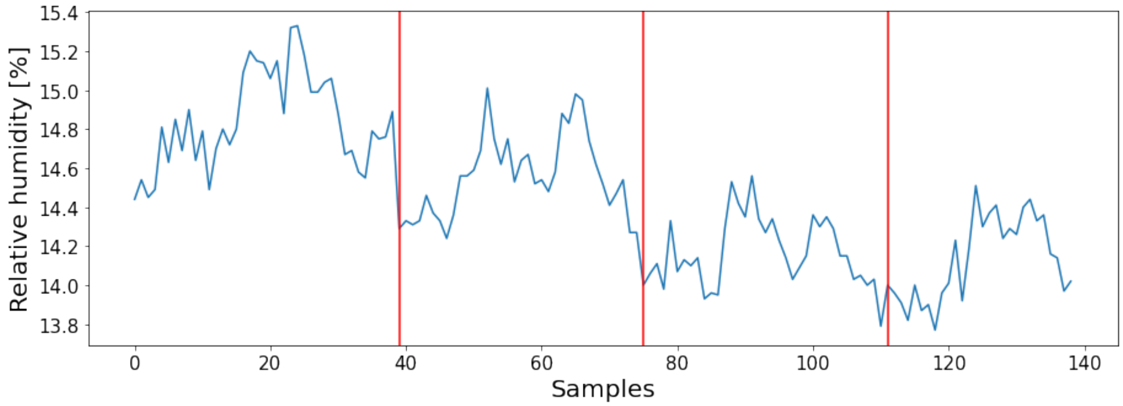

Figure 7: Humidity change during the measurements

Figure 7 shows humidity values during the measurements. The difference between maximum and minimum humidity values is 1.56%. The manufacturer of Olfactomics DMS stated that moisture change up to 2% RH does not cause noticeable effect on the measurement results. Again, red lines divide plot to well plates. As can be seen visually, the plot has slight negative trend with seasonal variations. We did not find yet explanation to this phenomena.

5 Distribution of classes

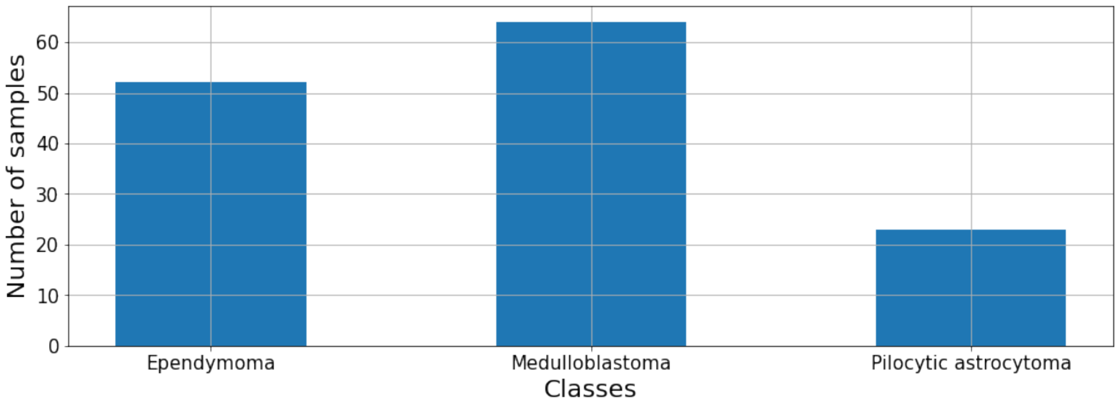

Figure 8: Distribution of classes in the data set

Distribution of classes (Figure 8) shows that there is a strong imbalance between the sample classes. Such imbalance coupled with small amount of data samples complicates the classification task significantly. With that amount of data, a classifier could simply assign all the samples to be, for example Medulloblastoma, and achieve an accuracy of approximately 46%, which would be greater than random guessing (33%).

One technique to mitigate the imbalance issue is using stratified cross-validation. The stratification ensures that each fold of data will contain approximately the same distribution of classes for training and testing.

6 Simple StratifiedGroupKFold cross-validation

The best classification result is achieved by using both channels, i.e. stacking the measured intensities of both positive and negative ions. After stacking, each dispersion plot is normalized with respect to the compensation voltage for the data to have the same scale. The scaling is needed because as the separation voltage increases the intensities decrease. Thus, each row in the dispersion plot has different scale. The last step in the data pre-processing is PCA transformation. The PCA transformation gives the best classification results with 50 components explaining over 88% of the variability in the original data. The plot showing accuracy depending on the number of PCA components is given in Figure 9. The red dotted line indicates the optimum at 50 components.

The algorithm used for classification of samples is Linear Discriminant Analysis (LDA). For obtaining stable classification result, training and testing of classifiers is repeated 100 times using a *for*

loop. The data is PCA transformed inside the loop. According to the sklearn documentation of PCA implementation, if a dataset contains too many features then randomness is included into the algorithm (for more details please refer to [2] and [3]). Also, the cross-validation object is created inside the loop for maintaining randomization.

The cross - validation took into account patients by using GroupCross validation with stratification. This type of cross-validation means that the data from a single patient is included either in testing or training set. For more details refer to [4].

[14]:
```
from sklearn.discriminant_analysis import LinearDiscriminantAnalysis
from sklearn.model_selection import cross_val_score, GroupKFold,␣
↪LeavePGroupsOut, LeaveOneGroupOut, GroupShuffleSplit
from sklearn.model_selection import StratifiedGroupKFold
from sklearn.decomposition import PCA
from sklearn.preprocessing import StandardScaler
from sklearn.metrics import accuracy_score, confusion_matrix,␣
↪ConfusionMatrixDisplay, classification_report

labelset = {i:l for l,i in enumerate(data['Class'].unique())}
revlabelset = {val:key for key,val in labelset.items()}
groups = data['Sample'].apply(lambda val: int(val[:-1])).to_list()
labels = data['Class'].apply(lambda val: labelset[val]).to_list()

Xneg = np.array(data['intensity_bottom'].to_list())
Xpos = np.array(data['intensity_top'].to_list())
Xjoined = np.hstack((Xpos, Xneg)) # join together both positive and negative␣
↪readings
groups = data['Sample'].apply(lambda val: int(val[:-1])).to_list()
y = np.array(data['Class'].apply(lambda val: labelset[val]).to_list())

# prepare empty matrices for the data
Xcpos = np.zeros((Xpos.shape[0], 1200))
Xcneg = np.zeros((Xpos.shape[0], 1200))

for i in range(Xpos.shape[0]):
    samplep = np.flipud(Xpos[i,:].reshape(20,60))
    samplen = np.flipud(Xneg[i,:].reshape(20,60))
    samplep = np.apply_along_axis(lambda row: (row - np.mean(row))/np.std(row),␣
↪0, samplep)# standardize column-wise
    samplen = np.apply_along_axis(lambda row: (row - np.mean(row))/np.std(row),␣
↪0, samplen)# standardize column-wise
    Xcpos[i,:] = samplep.flatten()
    Xcneg[i,:] = samplen.flatten()

Xcjoined = np.hstack((Xcpos, Xcneg))
cv_scores = []
for j in range(100):
    #kfold = LeavePGroupsOut(n_groups=1)
```

```
    npca = PCA(n_components=50)
    Xnpp_pca = npca.fit_transform(Xcjoined)
    kfold = StratifiedGroupKFold(n_splits=10)
    clf = LinearDiscriminantAnalysis(solver='svd')
    scores = cross_val_score(clf, Xnpp_pca, y, cv=kfold, groups=groups)
    cv_scores.append(np.mean(scores))
print("Avg accuracy score: {:.2f}%".format(np.mean(cv_scores)*100))
print("Std accuracy scores: {:.2f}%".format(np.std(cv_scores)*100))

# check how many PCA components are  needed and plot PCA components VS cv accuracy
Xcjoined = np.hstack((Xcpos, Xcneg))
gcv_scores = []
for c in np.arange(5, 95, 5):
    cv_scores = []
    for j in range(100):
        #kfold = LeavePGroupsOut(n_groups=1)
        kfold = StratifiedGroupKFold(n_splits=10)
        npca = PCA(n_components=c)#, whiten=False)
        Xnpp_pca = npca.fit_transform(Xcjoined)
        clf = LinearDiscriminantAnalysis(solver='svd')
        scores = cross_val_score(clf, Xnpp_pca, y, cv=kfold, groups=groups)
        cv_scores.append(np.mean(scores))
    gcv_scores.append(np.mean(cv_scores))
```

```
Avg accuracy score: 69.59%
Std accuracy scores: 1.87%
```

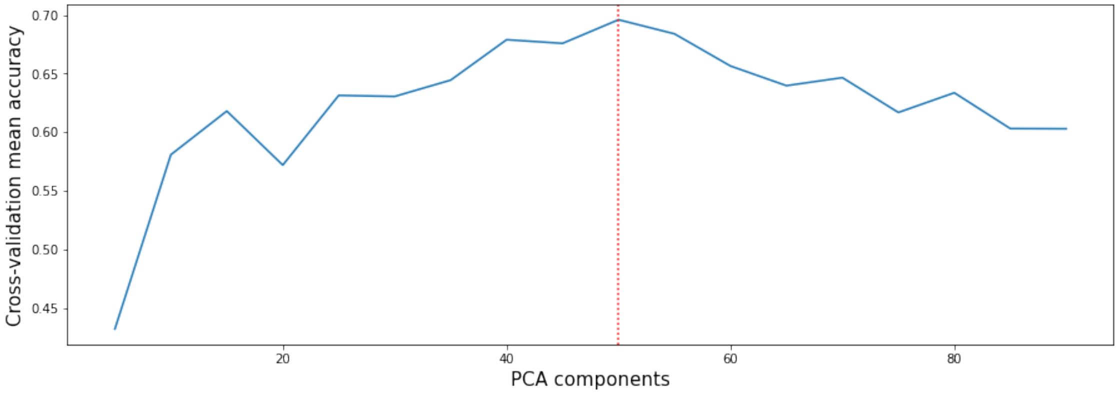

Figure 9: Selection of number of PCA components

7 Visualization of the previous cross-validation

[16]:
```
#kfold = LeavePGroupsOut(n_groups=1)
kfold = StratifiedGroupKFold(n_splits=10)
trlengths = []
tslengths = []
trlen_scaled = []
tslen_scaled = []
accs = []
counter = 1
for tri, tsi in kfold.split(Xcjoined, y, groups=groups):
    Xtrain, Xtest, ytrain, ytest = Xcjoined[tri], Xcjoined[tsi], y[tri], y[tsi]
    pca = PCA(n_components=50)
    Xtrain = pca.fit_transform(Xtrain)
    Xtest = pca.transform(Xtest)
    clf = LinearDiscriminantAnalysis(solver='svd')
    scores = cross_val_score(clf,Xtrain, ytrain)
    trlengths.append(len(tri))
    tslengths.append(len(tsi))
    accs.append(np.mean(scores))
    counter += 1

for i in range(counter-1):
    f = lambda x,up: up * (x/139)
    trlen_scaled.append(f(trlengths[i], accs[i]))
    tslen_scaled.append(f(tslengths[i], accs[i]))

fig, ax = plt.subplots(figsize=(20,10))
ax.bar(np.arange(1,counter), tslen_scaled, label='Test set')
ax.bar(np.arange(1,counter), trlen_scaled, bottom=tslen_scaled, alpha=0.5,␣
 ↪label='Train set')

_ = ax.bar_label(ax.containers[0], labels=tslengths, label_type='center',␣
 ↪size=20, color='white')
_ = ax.bar_label(ax.containers[1], labels=trlengths, label_type='center',␣
 ↪size=20)
_ = ax.set_xticks(np.arange(1,counter))
ax.set_xlabel('Cross-validation partition', fontsize=20)
ax.set_ylabel('Classification accuracy', fontsize=20)
ax.tick_params(axis='x', which='major', labelsize=24)
ax.tick_params(axis='y', which='major', labelsize=24)
ax.axhline(np.mean(accs), color='red', label='Mean accuracy', lw=3)
ax.grid(True)
_ = ax.legend(bbox_to_anchor=(0., 1.02, 1., .102), loc='lower left',
                      ncol=3, mode="expand", borderaxespad=0., fontsize=20)
```

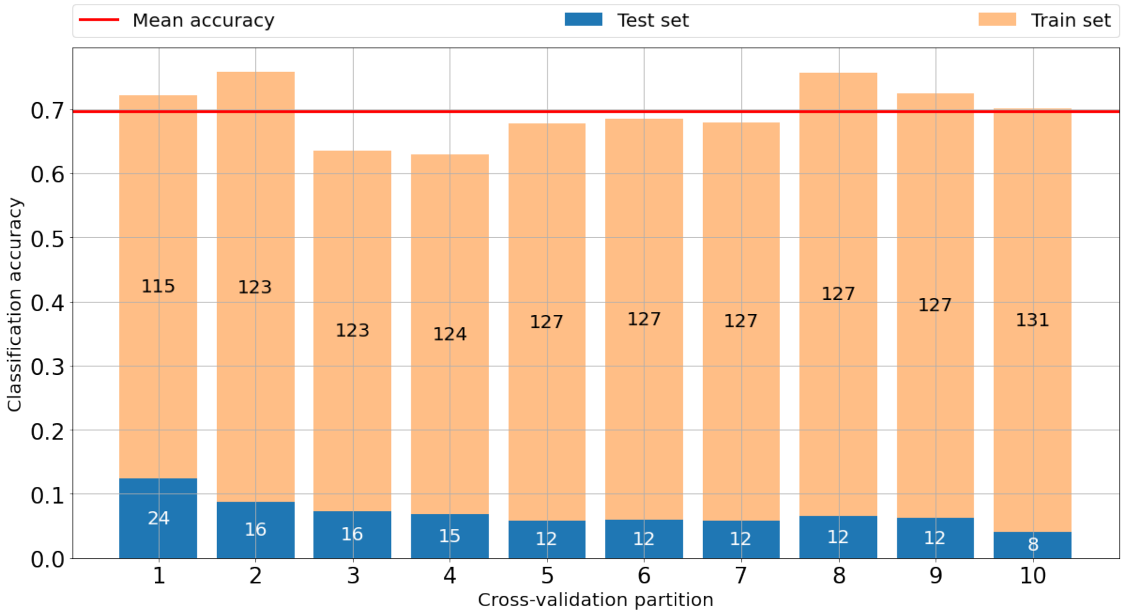

Figure 10: Cross-validation folds. The height of each bar represents classification accuracy.

As can be seen from Figure 10, the achieved mean accuracy is almost 70%. The size of test and training sets varied due to the use of cross-validation, which was explained earlier in this section.

8 Mean confusion matrix and classification report

Confusion matrix for all predictions during the cross-validation

[18]:
```
from sklearn.model_selection import cross_val_score, LeavePGroupsOut,␣
↪LeaveOneGroupOut, StratifiedGroupKFold
#kfold = LeavePGroupsOut(n_groups=1) # leave one group out
kfold = StratifiedGroupKFold(n_splits=10)
scores = []
tpreds = []
ttests = []
counter = 1
for tri, tsi in kfold.split(Xcjoined, y, groups=groups): # generator for train/
↪test indices
    Xtrain, Xtest, ytrain, ytest = Xcjoined[tri], Xcjoined[tsi], y[tri], y[tsi]␣
↪# split the data into train/test
    pca = PCA(n_components=50)
    Xtrain = pca.fit_transform(Xtrain) # apply PCA
    Xtest = pca.transform(Xtest) # apply PCA
    clf = LinearDiscriminantAnalysis(solver='svd')
    clf.fit(Xtrain, ytrain) # fit the classifier
```

```python
    preds = clf.predict(Xtest) # predict values in the test set
    tpreds += list(preds) # add predictions of the test set into the array
    ttests += list(ytest) # add right answers to the array
    accscore = accuracy_score(preds, ytest) #
cfmat = confusion_matrix(ttests, tpreds, normalize='pred') # calculate the confusion matrix through all the iterations
nncfmat = confusion_matrix(ttests, tpreds) # calculate the confusion matrix through all the iterations
short_label_set = {name[:6]:idx for name, idx in labelset.items()} # for displaying labels
disp=ConfusionMatrixDisplay(cfmat, display_labels=short_label_set)
nndisp = ConfusionMatrixDisplay(nncfmat, display_labels=short_label_set)
```

[19]:
```python
fig, ax = plt.subplots(1,2,figsize=(15,12), dpi=100)
plt.subplots_adjust(bottom=0.5, right=0.6, top=0.9, wspace=1)
disp=ConfusionMatrixDisplay(cfmat, display_labels=short_label_set)
nndisp = ConfusionMatrixDisplay(nncfmat, display_labels=short_label_set)
ax[0].set(title='Confusion Matrix Normalized')
ax[1].set(title='Confusion Matrix Not Normalized')
disp.plot(ax=ax[0],colorbar=False)
nndisp.plot(ax=ax[1], colorbar=False)
```

[19]:
```
<sklearn.metrics._plot.confusion_matrix.ConfusionMatrixDisplay at
0x7fbbd209fb80>
```

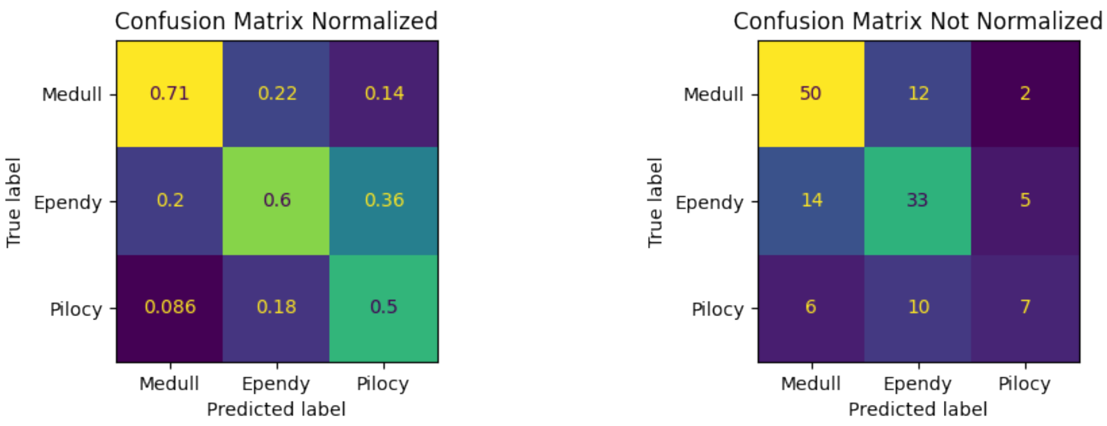

Figure 11: Confusion matrices with the classification results

The two confusion matrices (CM) in Figure 11 demonstrate classification performance for each class. The left CM is normalized with respect to the predicted label, which means that summing each column gives 1. Thus, the left CM shows classification probabilities. For example, the “Medull”-column has 3 values: Medull - Medull 0.71, Medull - Ependy - 0.20 and Medull - Pilocy 0.09. These

values mean that the class “Medull” is classified as being “Medull” with probability 0.71, as being “Ependy” with probability 0.20, and as being “Pilocy” with 0.09.

The right CM shows absolute classification results. For example, in case of Medulloblastoma (“Medull”), there are 50 samples of Medulloblastoma classified as Medulloblastoma, 12 as Ependymoma, and 2 sample as Pilocytic astrocytoma.

9 ROC/AUC curves

[21]:
```
from sklearn.metrics import roc_auc_score, roc_curve, auc
from sklearn.multiclass import OneVsRestClassifier
from sklearn.preprocessing import label_binarize
from sklearn.model_selection import cross_val_predict
from itertools import cycle
inv_labelset = {i:j for j,i in labelset.items()}

kfold = LeavePGroupsOut(n_groups=1) # leave one group out
#kfold = StratifiedGroupKFold(n_splits=10)

bin_labels = label_binarize(y, classes=[0,1,2])
clf = OneVsRestClassifier(LinearDiscriminantAnalysis(solver='svd'))
y_score = cross_val_predict(clf, Xjoined, bin_labels, cv=kfold,␣
 ↪method='predict_proba', groups=groups)

#
plt.figure(figsize=(20,10))
fpr = dict()
tpr = dict()
roc_auc = dict()
for i in range(3):
    fpr[i], tpr[i], _ = roc_curve(bin_labels[:, i], y_score[:, i])
    roc_auc[i] = auc(fpr[i], tpr[i])
colors = cycle(['blue', 'red', 'green'])
for i, color in zip(range(3), colors):
    plt.plot(fpr[i], tpr[i], color=color, lw=2,
             label='ROC curve of class {0} (auc = {1:0.2f})'
             ''.format(inv_labelset[i], roc_auc[i]))
plt.plot([0, 1], [0, 1], 'k--', lw=2)
plt.xlim([-0.01, 1.0])
plt.ylim([0.0, 1.01])
plt.xlabel('False Positive Rate', fontsize=20)
plt.ylabel('True Positive Rate', fontsize=20)
plt.xticks(fontsize=15)
plt.yticks(fontsize=15)
plt.legend(loc="lower right", fontsize=20)
plt.show()
```

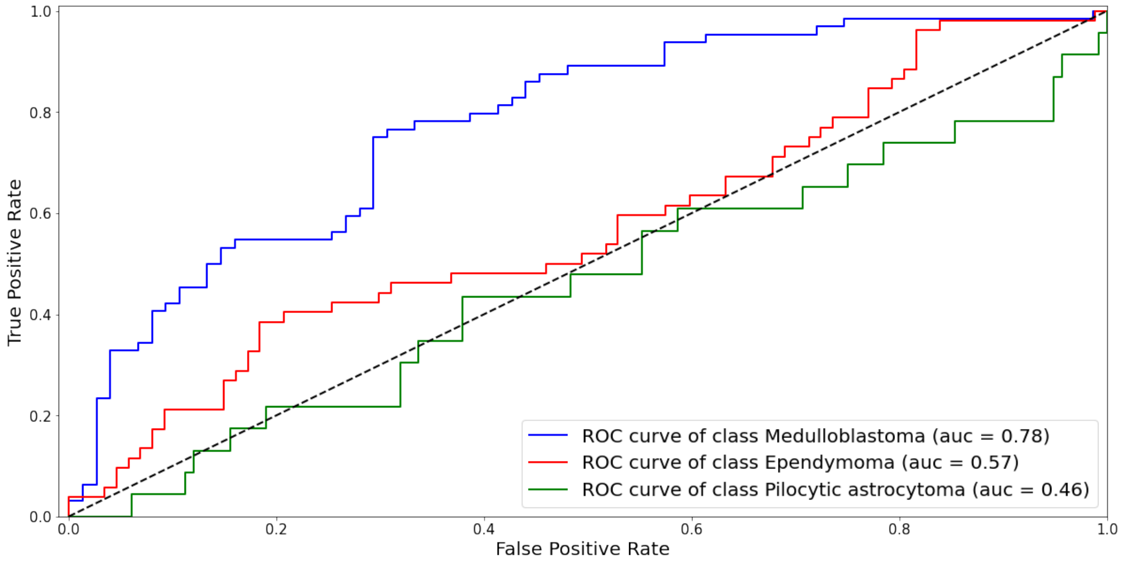

Figure 12: ROC/AUC curves for each class

Reciever Operating Characteristics (ROC) is an important metric for classification algorithms performance. In an ideal situation, the ROC curve goes up, vertically along the y-axis and at the top goes horizontally to the top-right corner. In this ideal case the Area Under the Curve (AUC) is 1. If the curve follows the dashed line, this means that the acuracy is close to guessing.

The ROC/AUC curves (Figure 12) demonstrate that only Medulloblastoma has somewhat noticable classification accuracy (auc=0.78). The Ependymoma has classification accuracy slightly better than guessing and Pilocytic astrocytoma follows almost perfectly guessing rate. Due to small amount of samples, it is impossible to be certain whether the classifier is unable to differentiate between the classes. As shown in Figure 8, the Medulloblastoma has the most samples in the data set, while Picotyic astrocytoma has slightly over 20 samples. The ROC/AUC behavior can be partly explained by the lack of data.

9.1 ML classification report (precision, recall)

Table 1: Classification report

	precision	recall	f1-score	support
**Medulloblastoma**	0.714	0.781	0.746	64
**Ependymoma**	0.600	0.635	0.617	52
**Pilocytic astrocytoma**	0.500	0.304	0.378	23
**accuracy**	0.648			
**macro avg**	0.605	0.573	0.580	139
**weighted avg**	0.636	0.648	0.637	139

Table 1 shows comprehensive information about the classification results. All four performance measurements are based on true positive (TP), false positive (FP), true negative (TN), and false

negative (FN) or a selection of those four. The terms used in the table are:

- precision
  - calculated with: TP / (TP + FP)
  - Intuitively it means that when the model predicts Medulloblastoma, it is correct 73% of time.
- recall
  - calculated with: TP / (TP + FN)
  - Intuitively, the model identifies correctly 75% of Medulloblastomas
- f1-score
  - calculated with: (precision * recall) / (precision + recall)
  - Harmonic mean of precision and recall.
- support
  - How many samples of the class was in the test set
- macro avg (macro average)
  - simple average of per-class f1-scores
- weighted avg
  - average of per-class f1-scores weighted with supports

9.2 Med classification report (sensitivity, specificity)

Researcher in the field of medicine often use terms sensitivity and specificity instead of precision and recall. Sensitivity is a special case of recall. Simply, sensitivity can be interpreted as: rate of correct identification of those who have a disease. The term specificity is interpreted as: rate of correct identification of those who do not have a disease.

$$sensitivity = \frac{TP}{TP + FN}$$

$$specificity = \frac{TN}{FP + TN}$$

[23]:
```
confmat = confusion_matrix(ttests, tpreds)
FP = np.sum(confmat, axis=0) - np.diag(confmat)
FN = np.sum(confmat, axis=1) - np.diag(confmat)
TP = np.diag(confmat)
TN = np.sum(confmat) - (FP + FN + TP)
sens = TP/(TP + FN) # identify correctly those who have disease
spec = TN/(FP + TN) # identify correctly those who don't have disease
df = pd.DataFrame({"Specificity":spec, "Sensitivity":sens})
df.index = ['Medulloblastoma', 'Ependymoma','Pilocytic astrocytoma']
df
```

Table 2: Classification report: sensitivity and specificity

	Medulloblastoma	Ependymoma	Pilocytic astrocytoma
**Specificity**	0.733	0.747	0.940
**Sensitivity**	0.781	0.635	0.304

10 Bonferroni correction

The Bonferroni correction method is used for reducing Type I error (false positives). Although opinions on the this method in the scientific community vary, it is here used for visualizing statistically significant differencies.

For visualizing differencies, this method should be applied pairwise. In our case pairwise means: Medulloblastoma VS Ependymoma, Ependymoma VS Pilocytic astrocytoma and Pilocytic astrocytoma VS Medulloblastoma.

[26]:
```
from scipy.stats import ks_2samp
pairs = [('Medulloblastoma', 'Ependymoma'), ('Medulloblastoma', 'Pilocytic␣
↪astrocytoma'),
        ('Ependymoma', 'Pilocytic astrocytoma')]

results = {}
for pair in pairs:
    # extract data
    data1 = data[data['Class'] == pair[0]]
    data2 = data[data['Class'] == pair[1]]
    X1 = np.zeros((len(data1), 20, 60))
    X2 = np.zeros((len(data2), 20, 60))
    bonferroni_image = np.zeros((20, 60))
    # prepare data: reshape, etc
    for i in range(len(data1)):
        sample = np.flipud(np.array(data1.iloc[i]['intensity_top']).
↪reshape(20,60))
        X1[i,:,:] = sample
    for i in range(len(data2)):
        sample = np.flipud(np.array(data2.iloc[i]['intensity_top']).
↪reshape(20,60))
        X2[i,:,:] = sample
    # go through each pair of pixel sets
    for i in range(20):
        for j in range(60):
            sample1 = X1[:,i,j]
            sample2 = X2[:,i,j]
            res = ks_2samp(sample1, sample2) # perform kolmogorov-smirnov 2␣
↪sample test
            if res[1] < 0.05/(len(sample1) + len(sample2)): # if p-value less␣
↪than length(sample) + length(sample2)
                bonferroni_image[i,j] = 1 # fill pixel coordinate with 1
    results[pair] = bonferroni_image # save the bonferroni image
```

[27]:
```
fig, ax = plt.subplots(3,1, figsize=(20,10))
for i,key in enumerate(results):
    ax[i].imshow(results[key], cmap='gray_r')
    ax[i].set_title("Bonferroni correction: " + key[0] + " vs " + key[1])
```

```
plt.tight_layout()
```

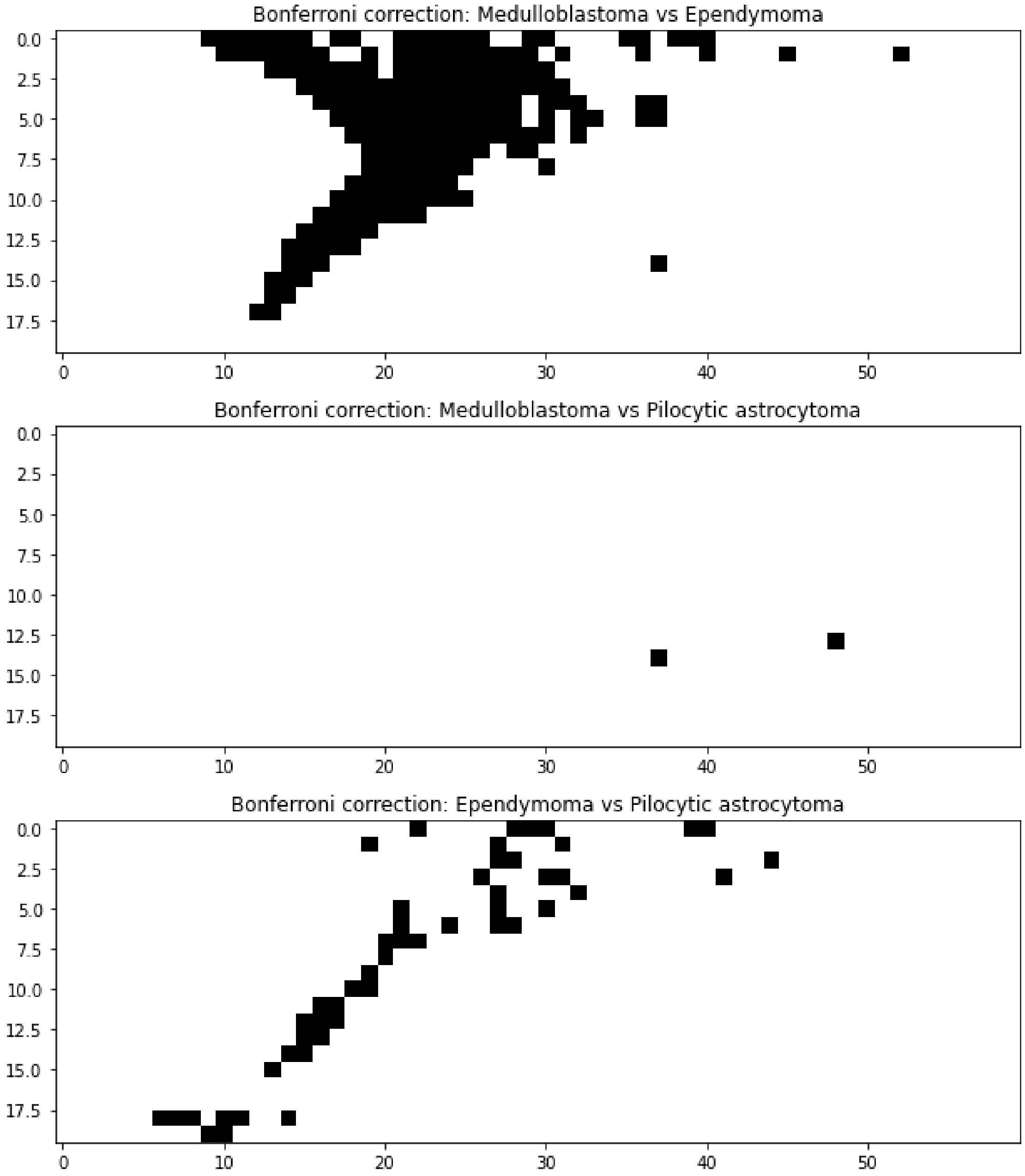

Figure 13: Results of Bonferroni corrections for each class

The three plots in Figure 13 demonstrate that:

1) Medulloblastoma differs significantly from Ependymoma

2) Medulloblastoma is statistically highly similar to Pilocytic astrocytoma

3) Ependymoma differs from Pilocytic astrocytoma

However, it does not mean that we have to measure only pixels designated by the Bonferroni correction method. In this case it only visualize differencies between classes.

11 Medulloblastoma. Subtyptes.

Vairous subtypes of medulloblastoma exist. Distinguishing reliably between these subtypes is impossible due to small amount of samples (presented below). To obtain meaningful results we need to use group cross-validation. The group cross-validation ensures that samples from the same patient do not appear in the train and test sets simultaneously. Thus, due to small amount of samples at each train/test iteration we end up with too small sizes of folds. For example, if the patient 10 is left for testing then we have only 28 samples for training.

[28]:
```
from scipy.stats import ks_2samp
medul = data[(data['Class'] == 'Medulloblastoma') & ~(data['Subclass'] ==␣
↪'Undefined')]
groups = medul.Sample.apply(lambda x: int(x[:-1])).to_list()
labels = medul['Subclass'].to_list()
X = np.array(medul['intensity_top'].to_list())
names = {name:i for i,name in enumerate(medul['Subclass'].unique())} # dict with␣
↪unique label names
labels = [names[i] for i in labels] # convert labels to 0 and 1
print("SHH number of samples",sum(np.array(labels) == 0))
print("non-WNT/non-SHH number of samples",sum(np.array(labels) == 1))
groups_info = {i:sum(np.array(groups) == i) for i in np.unique(np.array(groups))}
print()
for key, val in groups_info.items():
    print("Group", key, "has", val, "samples")
```

```
SHH number of samples 20
non-WNT/non-SHH number of samples 20

Group 10 has 12 samples
Group 14 has 4 samples
Group 17 has 16 samples
Group 19 has 8 samples
```

With that amount of data it is impossible to perform any reliable classification.

11.1 Bonferroni correction

[29]:
```
zerotype = np.apply_along_axis(lambda x: np.flipud(x.reshape(20, 60)), 1, X[np.
↪where(np.array(labels) == 0)[0],:])
onetype = np.apply_along_axis(lambda x: np.flipud(x.reshape(20, 60)), 1, X[np.
↪where(np.array(labels) == 1)[0],:])
bonferroni_image = np.zeros((20, 60))
for i in range(20):
```

```
    for j in range(60):
        s1 = zerotype[:,i,j]
        s2 = onetype[:,i,j]
        res = ks_2samp(s1, s2) # perform kolmogorov-smirnov 2 sample test
        if res[1] < 0.05/(len(s1) + len(s2)):
            bonferroni_image[i,j] = 1
plt.figure(figsize=(10,5))
plt.imshow(bonferroni_image, cmap='gray_r')
```

[29]: <matplotlib.image.AxesImage at 0x7fbbd3068d00>

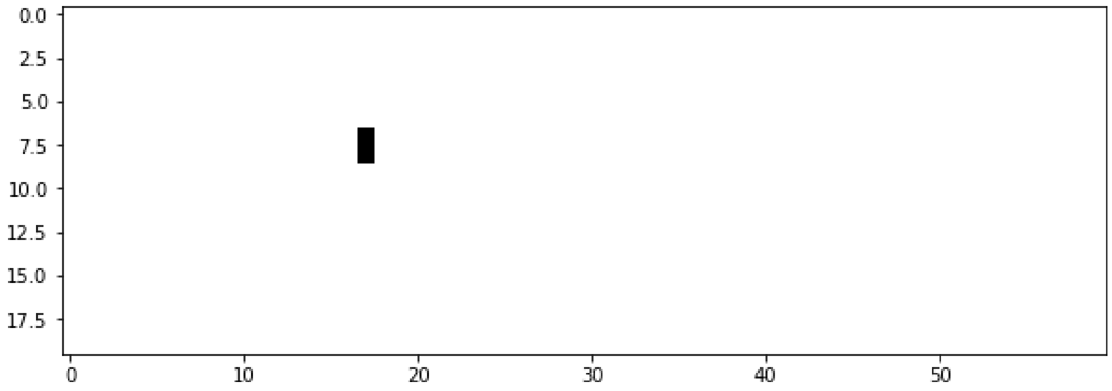

Figure 14: Results of Bonferroni correction for medulloblastoma subtypes

According to the Bonferroni correction, there are only 2 pixels that are statistically significant. The Bonferroni correction is done with the Kolmogorov-Smirnov two sample test. As stated above, the Bonferroni method is a questionable technique and used here only for visualization purposes. Thus, we can not draw reliable conclusions based on this plot.

11.2 PCA

[30]:
```
from sklearn.decomposition import PCA
from sklearn.preprocessing import StandardScaler
pca = PCA(n_components=0.99)
pca95 = PCA(n_components=0.95)
Xnorm = StandardScaler().fit_transform(X)
Xpca = pca.fit_transform(Xnorm)
Xpca95 = pca95.fit_transform(Xnorm)
plt.figure(figsize=(10,5))
plt.scatter(Xpca[:,0], Xpca[:,1], c=labels)
plt.title("First two PCA components", fontsize=25)
plt.xlabel("$PCA_1$", fontsize=15)
plt.ylabel("$PCA_2$", fontsize=15)
```

```
print("PCA explained 99% of total variation in the original data with the␣
↪first", pca.n_components_, "components")
print("PCA explained 95% of total variation in the original data with the␣
↪first", pca95.n_components_, "components")
```

```
PCA explained 99% of total variation in the original data with the first 38
components
PCA explained 95% of total variation in the original data with the first 31
components
```

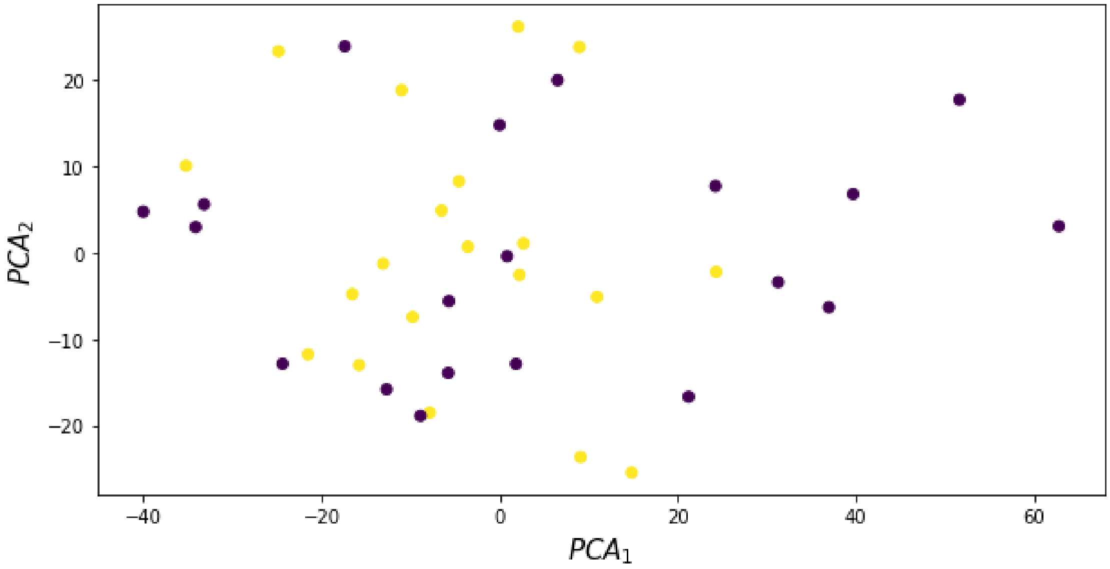

Figure 15: Scatter plot showing the first two PCA components

There is also no clear decision boundary between samples after PCA transformation. The PCA transformation on the plot (Figure 15) explained over 59.72% of variability.

12 Medulloblastoma against other types

The purpose of this part of analysis is to examine if machine learning algorithms can distinguish medulloblastoma from other types of tumors. For the analysis, the data set was divided into medulloblastoma samples (label 0) and other types of tumor samples (label 1). After applying GridSearch with different algorithms and data preprocessing techniques, the LDA was found to be the most accurate. The data set is normalized with respect to the compensation voltage and principal component analyisis is not applied. The average cross-validation score was 77.54%. For this configuration of the data set the LeavePGroupsOut cross-validation used with parameter P=3. The parameter P means that all combinations of three patients are only used for testing, not for training the classifier. Thus, this cross-validation technique requires more iterations yielding more stable results. Applying it to the data set configuration described in the previous sections did not

affect accuracy. Also, taking into account that this section tries to solve a binary classification problem, leaving 3 groups out of training makes test sets more diverse.

Table 3 shows the classification results during cross-validation. The accuracy metric is not enough for describing performance of the classifier trained and tested on heavily imbalanced data set. The table also shows precision and recall metrics. As can be seen from confusion matrix (Figure 16a), when the classifier predicts medulloblastoma it is correct 73% of times. If other type is predicted then it is correct 77% of times. According to the recall metric, the classifier accurately predicts 73% of all medulloblastoma samples and 77% of all samples of other types. The ROC curve in Figure 17 shows performance of the classifier at all classification thresholds. The ROC/AUC metric is useful when the data set is imbalanced.

[150]:
```
data2 = data.copy()
data2.loc[data2['Class'] != 'Medulloblastoma', 'Class'] = 'Other'
data2['Sample'] = data2['Sample'].apply(lambda row: str(row)[:-1])
groups = np.array([int(i) for i in data2['Sample'].to_list()])
labels = np.array([0 if i == 'Medulloblastoma' else 1 for i in data2['Class']])

Xneg = np.array(data2['intensity_bottom'].to_list())
Xpos = np.array(data2['intensity_top'].to_list())
Xjoined = np.hstack((Xpos, Xneg)) # join together both positive and negative␣
↪readings

# prepare empty matrices for the data
Xcpos = np.zeros((Xpos.shape[0], 1200))
Xcneg = np.zeros((Xpos.shape[0], 1200))

for i in range(Xpos.shape[0]):
    samplep = np.flipud(Xpos[i,:].reshape(20,60))
    samplen = np.flipud(Xneg[i,:].reshape(20,60))

    samplep = np.apply_along_axis(lambda row: (row - np.mean(row))/np.std(row),␣
↪0, samplep)# standardize column-wise
    samplen = np.apply_along_axis(lambda row: (row - np.mean(row))/np.std(row),␣
↪0, samplen)# standardize column-wise
    Xcpos[i,:] = samplep.flatten()
    Xcneg[i,:] = samplen.flatten()

Xcjoined = np.hstack((Xcpos, Xcneg))
raw_scores = []

n_components = 0
test_data_set = Xcjoined

for j in range(1):
    if n_components != 0:
        mmpca = PCA(n_components=n_components)
        pX = mmpca.fit_transform(test_data_set)
```

```
    else:
        pX = test_data_set.copy()
    kfold = LeavePGroupsOut(n_groups=3)
    clf = LinearDiscriminantAnalysis(solver='svd')
    scores = cross_val_score(clf, pX, labels, cv=kfold, groups=groups)
    raw_scores += list(scores)
print(f"Mean cross-validation scores: {np.mean(raw_scores):.2f}, STD: {np.
 ↪std(raw_scores):.2f}")

preds = [] # collect predictions
answs = [] # collect test labels
predsp = [] # collect probability predictions for ROC/AUC

for i in range(1):
    kfold = LeavePGroupsOut(n_groups=3)
    counter = 0
    for tri, tsi in kfold.split(test_data_set, labels, groups=groups):
        Xtrain, Xtest, ytrain, ytest = test_data_set[tri,:], test_data_set[tsi,:
 ↪], labels[tri], labels[tsi]
        if n_components != 0:
            pca = PCA(n_components=n_components)
            Xtrain = pca.fit_transform(Xtrain)
            Xtest = pca.transform(Xtest)
        clf = LinearDiscriminantAnalysis(solver='svd')
        clf.fit(Xtrain, ytrain)
        y_pred = clf.predict(Xtest)
        y_predp = clf.predict_proba(Xtest)
        preds += list(y_pred.flatten())
        answs += list(ytest.flatten())
        predsp += list(y_predp[:,1].flatten())
```

```
Mean cross-validation scores: 0.78, STD: 0.15
```

Table 3: Classification report

	precision (%)	recall (%)	f1-score (%)	support
**Medulloblastoma**	73.3	72.8	73.1	5824
**Other types**	76.9	77.4	77.2	6825
**accuracy (%)**		75.3		
**macro avg (%)**	75.1	75.1	75.1	12649
**weighted avg (%)**	75.3	75.3	75.3	12649

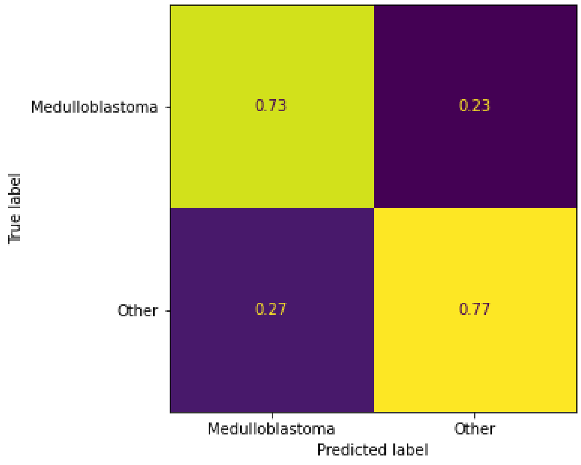

(a) Normalized confusion matrix

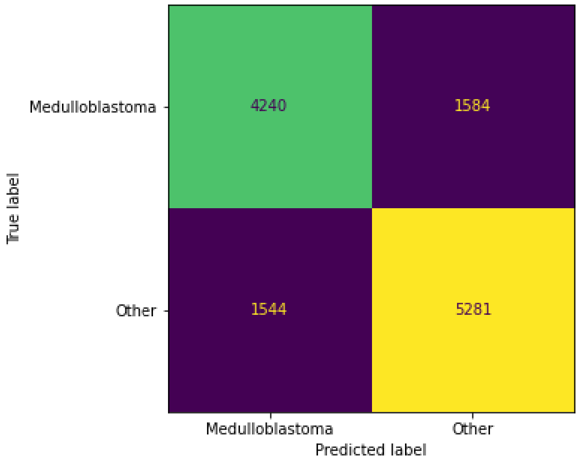

(b) Not normalized confusion matrix

Figure 16: Confusion matrices for the cross-validation

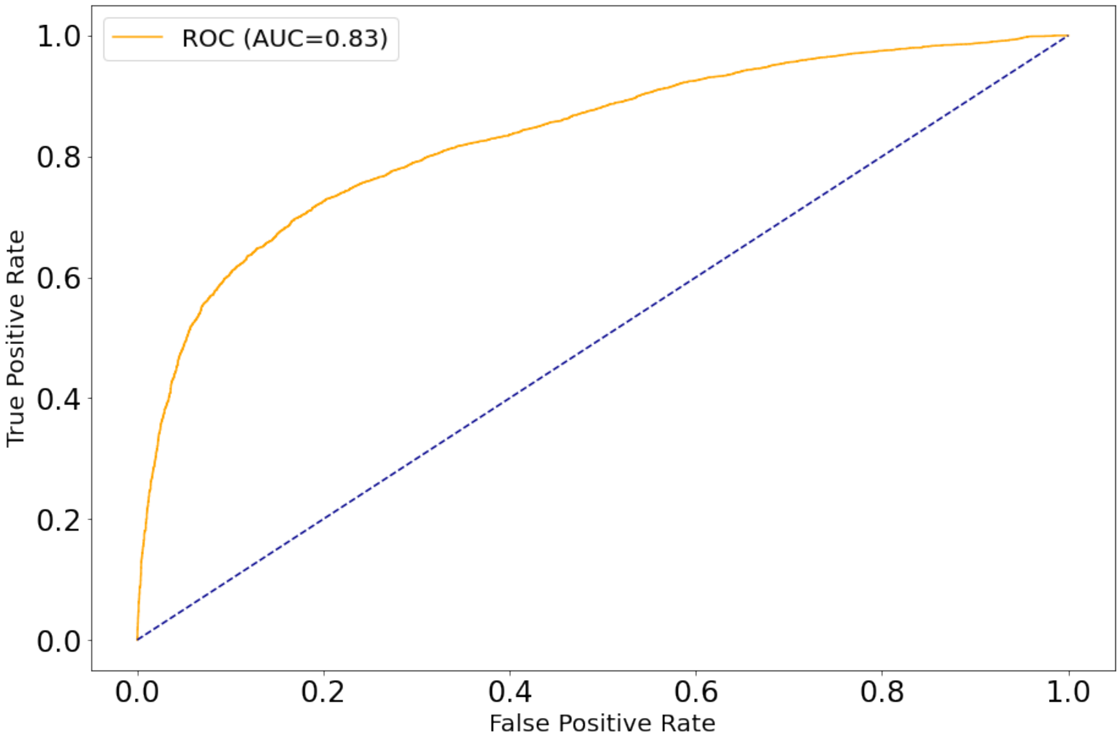

Figure 17: ROC-curve

Finally, images in Figure 18 demonstrates that there are no visual differences between dispersion

plots with medulloblastoma and other dispersion plots. However, the differences become visible at the upper-left part of the dispersion plot after subtracting the medulloblastoma mean dispersion plot from the mean dispersion plot of other types.

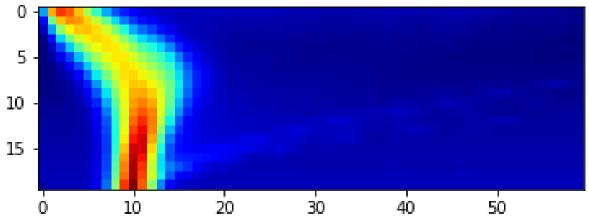

(a) Mean dispersion plot of medulloblastoma

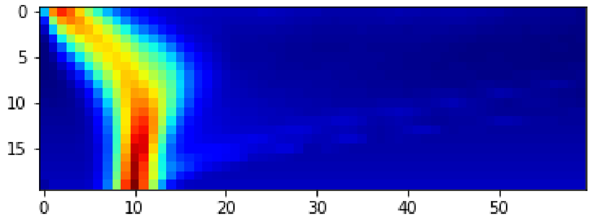

(b) Mean dispersion plot of other types

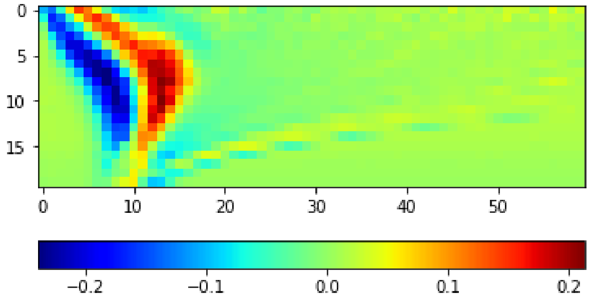

(c) Difference between mean dispersion plots

Figure 18: Differences between mean dispersion plots

References

[1] Z. Safaei et al. “Differential Mobility Spectrometry of Ketones in Air at Extreme Levels of Moisture”. In: *Scientific Reports* 9.1 (Dec. 2019). ISSN: 20452322. DOI: 10.1038/S41598-019-41485-7. (Visited on 04/01/2022).

[2] *PCA: scikit-learn documentation*. URL: https://scikit-learn.org/stable/modules/generated/sklearn.decomposition.PCA.html.

[3] *Finding Structure with Randomness: Stochastic Algorithms for Constructing Approximate Matrix Decompositions (Unpublished)*. URL: https://authors.library.caltech.edu/27187/.

[4] *Group Crossvalidation: scikit-learn documentation*. URL: https://scikit-learn.org/stable/modules/generated/sklearn.model_selection.StratifiedGroupKFold.html?highlight=stratified+group+kfold#sklearn.model_selection.StratifiedGroupKFold.
