# Supplementary material for: Rapid identification of pediatric brain tumors with differential mobility spectrometry
Source: Front Oncol. 2024 Apr 30;14:1352509. doi: 10.3389/fonc.2024.1352509 (PMC11091372; doi:10.3389/fonc.2024.1352509)
Supplement: Supplementary file 4 [file Table_2.docx]

| **Patient ID** | **Gender** | **Age (years)** | **Tumor location** | **Integrated diagnosis** | **Histology** | **Immunohistochemistry** | **Method for the genetic/epigenetic testing** | **Primary/recurrent** |
| --- | --- | --- | --- | --- | --- | --- | --- | --- |
| 1 | F | 8 | Supratentorial | Ependymoma grade 3 | Perivascular pseudorosettes, mitoses (7/10 HPF), necrosis, microvascular proliferation | GFAP+, CD56+, MAP-2+, synaptophysin+, neurophilament+, NEU-N+, P53 10%, EGFR-, INI-1+, MIB-1 20-30% | DNA methylation analysis – incongruity with histology | Primary |
| 2 | M | 1 | Supratentorial | Ependymoma grade 2 | Perivascular pseudorosettes, no mitoses, no necrosis, no microvascular proliferation | GFAP+, INI-1+, EMA+, IDH1-, synaptophysin-, p53-, MIB-1 9% | DNA methylation analysis – incongruity with histology | Primary |
| 3 | F | 1 | Infratentorial | Pilocytic astrocytoma grade 1 | Bipolar astrocytic cells, only few Rosenthal fibers, few mitoses (2/10 HPF), no necrosis, no microvascular proliferation | GFAP-, INI-1+, IDH1-, p53-, MIB-1 7% | Not performed | Primary |
| 4 | F | 7 | Infratentorial | Pilocytic astrocytoma grade 1 | Bipolar astrocytic cells, microcysts, Rosenthal fibres, no mitoses, no necrosis, no microvascular proliferation | GFAP+, BRAF+, INI-1+, IDH1-, p53-, MIB-1 < 1% | Not performed | Primary |
| 5 | F | 1 | Infratentorial | Ependymoma grade 3, PFA | Perivascular pseudorosettes, nuclear atypia, mitoses (8/10 HPF), necrosis, microvascular proliferation | GFAP+, EMA+, IDH1-, INI-1+, p53+, MIB-1 20% | DNA methylation analysis | Primary |
| 6 | M | 4 | Infratentorial | Ependymoma grade 3, PFA | Perivascular pseudorosettes, few mitoses (2/10 HPF), necrosis, microvascular proliferation | GFAP+, EMA+, INI-1+, p53-, IDH1-, BRAF-, MIB-1 15% | DNA methylation analysis | Primary |
| 7 | F | 15 | Infratentorial | Medulloblastoma grade 4, SHH, TP53 undefined | Classic variant, several mitoses, necrosis | synaptophysin+, NSE+, NeuN+, CD56+, S-100-, GFAP-, INI-1+, p53-, Gomori-, MIB-1 40%, beta-catenin+ | DNA methylation analysis, FISH | Primary |
| 8 | M | 6 | Supratentorial | Ependymoma grade 3, supratentorial with RELA -fusion | Perivascular pseudorosettes, mitoses (10/10 HPF), necrosis, no microvascular proliferation | GFAP+, vimentin+, S-100+, INI-1+, ATRX+, CD99+, EMA+, p53 50%, CD56+, MAP-2+, NeuN-, synaptophysin-, IDH1-, BRAF-, MIB-1 12% | DNA methylation analysis | Primary |
| 9 | M | 9 | Infratentorial | Medulloblastoma grade 4, group 3/4 | Classic variant, several mitoses, necrosis | synaptophysin+, MAP-2+, NeuN+, L1-CAM+, p53 5%, Ki-67 70%, GFAP-, INI-1+, IDH1-, BRAF-, EGFR-, Gomori+, beta-catenin-, YAP1-, GAB1- | DNA methylation analysis, FISH, NGS | Primary |
| 10 | M | 1 | Infratentorial | Medulloblastoma grade 4, SHH, TP53 wild type | Desmoplastic/nodular variant, mitoses (20/10 HPF), no necrosis | synaptophysin+, NSE+, NeuN+, Ki-67 70%, beta-catenin+, GFAP-, BRAF-, p53-, L1-CAM+, ATRX-, IDH1-, INI-1+, YAP1+, GAB1+ | DNA methylation analysis, FISH, NGS | Recurrent |
| 11 | M | 1 | Infratentorial | Pilocytic astrocytoma grade 1 | Bipolar astrocytic cells, Rosenthal fibres, no mitoses, no necrosis, no microvascular proliferation | GFAP+, p53-, INI-1+, ATRX+, BRAF-, V600E-, IDH1-, Ki-67 6% | DNA methylation analysis | Primary |
| 12 | F | 1 | Infratentorial | Medulloblastoma grade 4, SHH, TP53 wild type | Desmoplastic/nodular variant, several mitoses, no necrosis | synaptophysin+, NeuN+, GFAP-, YAP1+, GAB1+, INI-1+, p53-, IDH1-, BRAF-, V600E-, H3K27m-, CD34-, ATRX+, beta-catenin-, Ki-67 50% | DNA methylation analysis, NGS | Primary |
| 13 | M | 17 | Infratentorial | Pilocytic astrocytoma grade 1 | Bipolar astrocytic cells, Rosenthal fibres, microcysts, no mitoses, no necrosis, no microvascular proliferation | GFAP+, IDH1-, ATRX+, p53-, BRAF-, V600E-, Ki-67 4% | DNA methylation analysis | Primary |
| 14 | M | 7 | Infratentorial | Medulloblastoma grade 4, group 4 | Partially desmoplastic/nodular, partially classic variant, mitoses (25/10 HPF), Homer Wright rosettes, no necrosis | synaptophysin+, chromogranin A+, MAP2+, NeuN+, GFAP-, desmin-, CK7/8-, SOX10-, EMA-, CD34-, BRAF-, IHC-, ATRX+, H3 TRIME+, INI-1+, IDH1-, p53-, beta-catenin?, YAP1-, GAB1- | DNA methylation analysis, FISH | Primary |
| 15 | M | 10 | Supratentorial | Pilocytic astrocytoma grade 1 | Bipolar astrocytic cells, microcysts, no Rosenthal fibres, no necrosis, no microvascular proliferation | GFAP+, IDH1-, ATRX+, INI-1+, BRAF+, p53 4%, CD34-, Ki-67 5% | DNA methylation analysis | Recurrent |

Abbreviations: FISH, fluorescence in situ hybridization; NGS, next generation sequencing
